# Optical Characterization in Microelectronics Manufacturing

**DOI:** 10.6028/jres.099.058

**Published:** 1994

**Authors:** S. Perkowitz, D. G. Seiler, W. M. Duncan

**Affiliations:** National Institute of Standards and Technology, Gaithersburg, MD 20899-0001; Texas Instruments, Inc., Dallas, TX 75243

**Keywords:** ellipsometry, infrared spectroscopy, modulation spectroscopy, optical microscopy, photoluminescence, Raman scattering

## Abstract

To successfully construct semiconductor devices, the semiconductor industry must measure fundamental material parameters, especially when developing new materials; measure the quality of the material as it is grown; *accurately* determine the details of thin films, quantum wells, and other microstructures that control or affect device performance; and measure properties of the devices themselves. Properties that need to be determined, therefore, include basic band structure and transport parameters, such as energy gap values and carrier scattering times; the presence and concentration of impurities and defects; alloy parameters; layer thicknesses; the distribution of materials in complex structures; and many others. This process of determining a wide range of material, structural, and device parameters is called characterization. The semiconductor industry uses many characterization methods which draw on electrical, chemical, and other approaches. Among these, optical characterization techniques, defined as those using electromagnetic radiation from the ultraviolet to the far infrared, stand out because they arc nondestructive and require minimal sample preparation since no contacts arc needed. These features arc of great importance for production use or to examine finished devices. Another benefit is that, unlike electrical methods which require fixed contacts, optical techniques can give two- or three-dimensional maps of properties over the extent of a semiconductor wafer. The six techniques described in this paper (cllipsometry, infrared spectroscopy, microscopy, modulation spectroscopy, photolumincscence, and Raman scattering) were chosen because they are currently or potentially widely used in the industry; they measure a broad array of semiconductor parameters; and they operate in different regions of the electromagnetic spectrum. The discussion of each technique indicates the basic semiconductor quantities measured, gives the scientific basis of the technique, and indicates how the measurement is made. Illustrative examples from the literature are discussed in detail, showing applications to important semiconductor materials. More information can be obtained from the detailed list of references included.

## 1. Introduction

To successfully construct semiconductor devices, the microelectronics industry must measure fundamental material parameters, especially when developing new materials; measure the quality of the material as it is grown; accurately determine the structural details of thin films, quantum wells, and other microstructures at the heart of devices; and measure properties of the devices themselves. Properties that need to be determined, therefore, include basic band structure and transport parameters, such as gap values and carrier scattering times, the presence and concentration of impurities and defects, alloy parameters, layer thicknesses, the distribution of materials in complex structures, and many others.

The semiconductor industry uses many characterization methods which draw on electrical, chemical, and other approaches. Among these, optical characterization techniques, defined as those using electromagnetic radiation from the ultraviolet to the far infrared, stand out because they are nondestructive and require minimal sample preparation, since no contacts are needed. These features are of great importance for production use, for on-line applications, and for examination of finished devices. Another benefit is that optical techniques can give two- or three-dimensional maps of properties over the extent of a semiconductor wafer without requiring fixed contacts.

Six techniques are described in this paper (ellipsometry, infrared spectroscopy, optical microscopy, modulation spectroscopy, photoluminescence, and Raman scattering). They were chosen because they are currently widely used in the industry and because they measure a broad array of semiconductor parameters. The discussion of each technique indicates the basic semiconductor quantities measured (see Table 1), the physical basis of the technique, and how the measurement is made. Illustrative examples from the literature are discussed, showing applications to important semiconductor material systems. A more detailed review of infrared, Raman and photoluminescence spectroscopies is given in a book by Perkowitz [1]. A recent review of the optical properties of semiconductors is given by Amirtharaj and Seiler [2].

### 1.1 A Note on Units

Some regions of the electromagnetic spectrum and some optical methods, refer to wavelength as a matter of usage; others use wavenumbers, or photon energy. Each section here uses the most common units for that technique, including wavelength in nanometers (nm) and micrometers (μm); wavenumber in cm^−1^; and photon energy in electron volts (eV). Table 2 shows conversion factors for the main units of measure usually encountered.

### 1.2 References

[1] S. Perkowitz, Optical Characterization of Semiconductors: Infrared, Raman, and Photoluminescence Spectroscopy, Academic Press, London (1993).
[2] P. Amirtharaj and D. G. Seiler, Optical Properties of Semiconductors, Chapter in Handbook of Optics, McGraw-Hill, to be published.

## 2. Ellipsometry

### 2.1 Introduction

Ellipsometry is a technique widely used to measure the thicknesses of films important to semiconductor technology, such as SiO_2_ on Si. Thicknesses measured are typically in the range of several nm to several hundred nm. Surface cleanliness of semiconductor wafers during processing can also be determined. In spectroscopic ellipsometry, the ellipsometric data are obtained as a function of wavelength. Then appropriate modeling and fitting can yield the dielectric functions and thicknesses of the layers in complex semiconductor/oxide multilayer systems, such as SIMOX (Separation by IM-planted OXygen), a silicon-on-insulator material formed by high-energy oxygen ion implantation in silicon. The dielectric functions give a complete picture of composition for the entire layered structure.

### 2.2 Physical Basis

Ellipsometry is based on the polarization transformation that occurs when a beam of polarized light is reflected from (or transmitted through) an interface or film. For example, if plane- or linearly-polarized light impinges on the surface of an absorbing medium, the reflected light usually becomes elliptically polarized because the reflection proeess differently affects the in-plane component of the incident electric field *E*_p_, relative to the perpendicular electric field component *E*_s_. Eaeh component is reflected with new values of amplitude and phase. The key parameters obtained from an ellipsometrie measurement are the ellipsometrie angles *ψ* and *⊿.* These appear in the complex reflection ratio *ρ*, defined as

$$\rho =\frac{r_{p}}{r_{s}}=\tan\left(\psi \right)e^{i\Delta },$$ (1)

where the amplitude reflection coefficients *r*_p_ and *r*_s_ are

$$r_{p}=\frac{E_{p}\left(\text{reflected}\right)}{E_{p}\left(\text{incident}\right)}$$ (2)

$$r_{s}=\frac{E_{s}\left(\text{reflected}\right)}{E_{s}\left(\text{incident}\right)}.$$ (3)

The ellipsometric angles are defined as *ψ* = tan^−1^|*ρ*|, and *⊿* is the difference in phase between the p and s components.

### 2.3 Experimental and Technical Details

Ellipsometric measurements start with light of known polarization incident on the sample. The polarization of the reflected light is determined, from which further analysis gives the parameters such as refractive index and film thickness which determine the interaction between light and sample.

In its simplest form, single-wavelength ellipsometry requires a manual nulling to gather data. Light from the source (usually a laser for single-wavelength work) passes through a linear polarizer, then through a compensator which elliptically polarizes the light. The light continues to the sample, is reflected, passes through a polarization analyzer, and is finally detected. The null technique works by adjusting the angle of the polarizer with respect to compensator, sample, and analyzer until the reflection process just cancels the ellipticity the light gained from the compensator. Then the reflected light is linearly polarized and can he extinguished by choosing the appropriate angle for the analyzer. that is, until the photomultiplier shows a minimum signal. The two values of the angles yield *ρ*.

This null process is too slow for real-time measurements, or for spectroscopic ellipsometry. Three types of automatic ellipsometry (self-compensating, rotating element, and polarization-modulated), together with dedicated computers, allow rapid measurement and analysis. In the automatic elf-compensating system [1], the angles of the linearly polarized light leaving the polarizer, and enteringthe analyzer, arerotated by Faraday or Pockets cells, until the null is achieved. This type of instrument can give fixed wavelength data within 1 ms, and spectroscopic data over a wide wavelength range in 3 s.

**Fig. 1:**
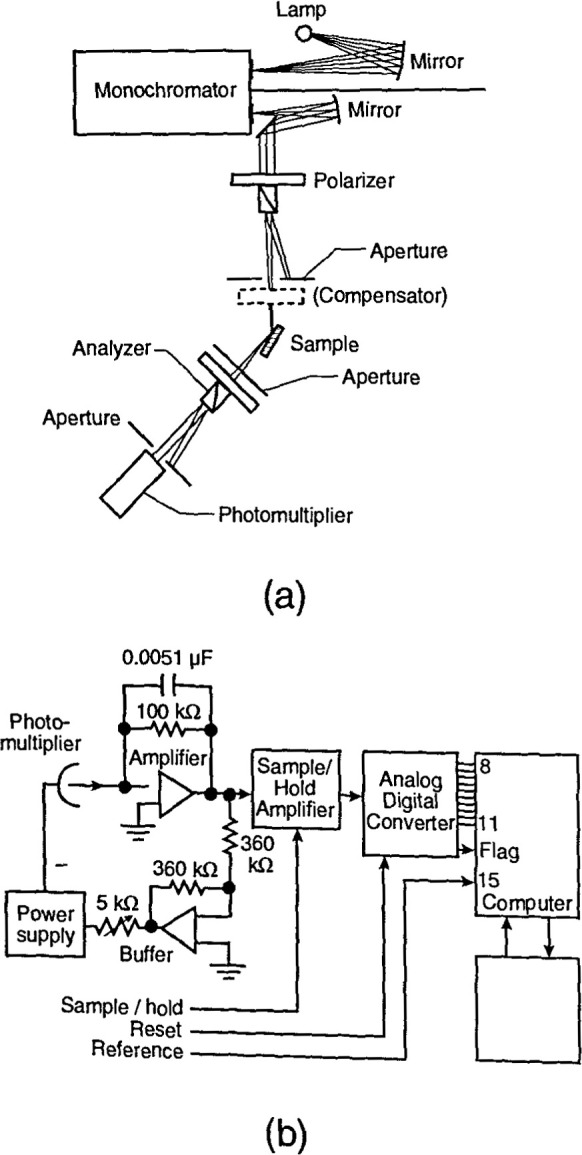
Schemalie diagrams of (a) optical elements and (b) signal processing system for a rotating analyzer spectroscopic ellipsometer designed for high-precision measurements of the optical properties of solids. (See See. 2.5, General Refs., Collins (1990), fig. 3, p.2032.)

The optical layout of the rotating element system (Fig. 1) is like that of the self-compensating system with the compensator omitted [1]. The polarization analyzer rotates around the axis of the reflected light beam at a fixed angular velocity, typically corresponding to 50 Hz to 100 hz. The rotating analyzer would produce a sine-squared signal with two maxima and two true zero minima every rotation, if the light were linearly polarized. For elliptically polarized light, the signal is also of sine-squared form, but with smaller maxima, and nonzero miniama, which depend on the ellipticity of the reflected light. A Fourier analysis of the output of the rotating analyzer gives *ρ* and hence the angles *ψ* and ⊿. For an analyzer rotating at 100 Hz, the measurement at a single wavelength can be completed in 5 ms.

The fastest type of system is the polarization-modulated ellipsometer [1], where the compensator in the manual null system is replaced by a birefringentphase modulator (a piezobirefringent plate or a Pockets cell). In the phase modulator, the ellipticity imparted to the linearly polarized light varies sinusoidally with time, rather than remaining constant as in the self-compensating system. The signal which results at the detector can be Fourier analyzed or analyzed by a phase-sensitive detector togive *ρ*. A piezobirefringent modulator is a fast device, which can operate at 100 kHz or more; hence, this system can obtain data in an interval of 10 ms per wavelength measurement, which means that full scans over the range 400 nm to 700 nm can be obtained in a few seconds or less.

Of the three automatic systems, the polarization-modulated spectrometer is best for real-time rapid data acquisition. However, in the self-compensating and polarization-modulation cases, the compensator or modulator must be tuned for each wavelength; hence, these are more complex and can be less accurate than the rotating analyzer system.

For spectroscopic ellipsometry, a stable xenon lamp with output covering the near ultraviolet to near infrared is a commonly used source. The sample is mounted on a high-accuracy stage to allow careful angle alignment. Usually an autocollimator and apertures are used to control collimation and alignment. In general, available equipment gives good results over the near infrared to the near ultraviolet. The ellipsometric angles *⊿* and *ψ* can be measured to within millidegrees, resulting in uncertainties of less than one part in 10^3^ for the index of refraction and tenths of a nanometer for the corresponding thicknesses.

Parameters for a complex semiconductor/oxide system examined by ellipsometry are determined by sophisticated computer software [1–2]. These fit the measured ellipsometric parameters versus wavelength, by assuming appropriate dielectric functions for each layer, and layer thicknesses. Commercial systems include appropriate software, and fitting routines are also available from other sources.

### 2.4 Illustrative Applications

**Fig. 2:**
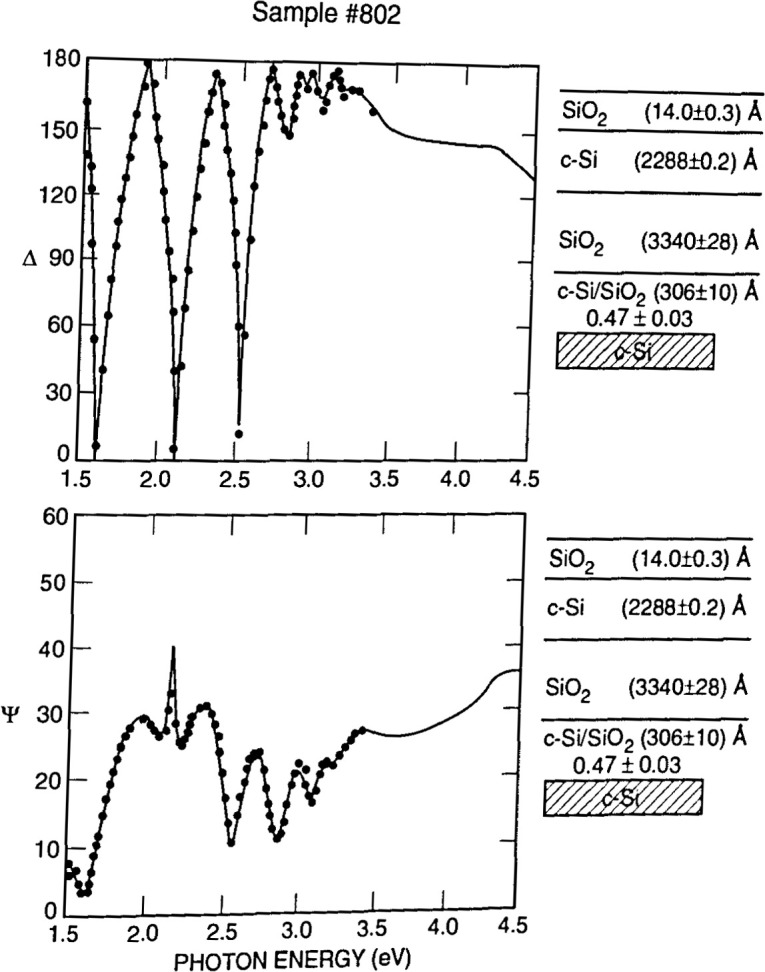
Ellipsometric angles ⊿ and *ψ*versus photon energy for a SIMOX sample. Individual points, data obtained from rotating element spectroscopic ellipsometer. Solid line, fit obtained by regression analysis assuming structural layer thickness and composition shown on the right. The layer immediately below the 334 nm SiO_2_ layer is modeled as a granular mixture of crystalline Si(e-Si) and SiO_2_, with 47% volume fraction c-Si, using effective medium theory. Shown to the right of the figures are the thickness of the layers. The calculated uncertainties of the model parameters are set to one standard deviation as determined by the regression analysis. (After D. Chandler-Horowitz et al. (1991), unpublished data, National Institute of Standards and Technology.)

An example of the kind of semiconductor analysis that can be achieved with spectroscopic ellipsometry is shown in Fig. 2 for a sample of SIMOX, an important silicon-on-insulator system. The ellipsometric angles *⊿* and *ψ* show complex spectra over the range 1.5 eV to 4.5 eV, with the large oscillations related to interference effects. Multiparameter regression analysis yields the fits displayed in the plots, which determine the sample’s structural details as shown.

Table 3 presents typical sensitivities of quantities obtained by ellipsometry, such as thicknesses, composition, and temperature. For more specific details, the reader can refer to the citations given in the table.

### 2.5 References

[1] O. Acher, E. Bigan, and B. Drevillon, Improvements of phase-modulated ellipsometry, Rev. Sci. Instrum. **60**, 65 (1989).
[2] R. M. A. Azzam and N. M. Bashara, Ellipsometry and Polarized Light, North-Holland, New York (1989).

#### General

D. E. Aspnes, The characterization of materials by spectroscopic ellipsometry, in Spectroscopic Characterization Techniques for Semiconductor Technology, Proceedings SPIE Vol. 452, F. H. Pollak and R. S. Bauer, eds., SPIE, Bellingham, Washington (1983) pp. 60–70.
D. E. Aspnes, The accurate determination of optical properties by ellipsometry, in Handbook of Optical Constants of Solids, E. D. Palik, ed., Academic Press, Orlando, Florida (1985) pp. 89–112.
D. E. Aspnes, Analysis of semiconductor materials and structures by spectroellipsometry, in Spectroscopic Characterization Techniques for Semiconductor Technology III, Proceedings SPIE Vol. 946, O. J. Glembocki, F. H. Pollak, and F. Ponce, eds., SPIE, Bellingham, Washington (1988) pp. 84–97.
R. W. Collins, Automatic rotating element ellipsometers: calibration, operation, and real-time applications, Rev. Sci. Instrum. **61**, 2029–2062 (1990).
J. F. Marchiando, Semiconductor Measurement Technology: A Software Program for Aiding the Analysis of Ellipsometric Measurements, Simple Spectroscopic Models, Natl. Inst. Stand. Technol. Special Publication 400-84, U.S. Government Printing Office, Washington, DC (1990).
B. A. Tirri, A. Turner, and P. C. Van Buskirk, Spectroellipsometric characterization of inhomogencous films, in Modern Optical Characterization Techniques for Semiconductors and Semiconductor Devices, Proceedings SPIE Vol. 794, O. H. Glembocki, F. H. Pollak and J. J. Soong, eds., SPIE, Bellingham, Washington (1987) pp. 252–261.

#### Applications

D. E. Aspnes and A. A. Studna, Optical detection and minimization of surface overlayers on semiconductors using spectroscopic ellipsometry, in Optical Characterization Techniques for Semiconductor Technology, Proceedings SPIE Vol. 276, D. E. Aspnes, S. So, and R. F. Potter, eds., SPIE, Bellingham, Washington (1981) pp. 227–232.
D. E. Aspnes, J. P. Harbison, A. A. Studna, L. T. Flotez, and M. K. Kelly, In situ optical measurements of the growth of GaAs and AIGaAs by molecular beam epitaxy, in Spectroscopic Characterization Techniques for Semiconductor Technology III, Proceedings SPIE Vol. 946, O. J. Glembocki, F. H. Pollak, and F. Ponce, eds., SPIE, Bellingham, Washington (1988) pp. 112–121.
R. W. Collins and J. M. Cavese, In situ ellipsometry characterization of the growth of thin film amorphous semiconductors, in Modern Optical Characterization Techniques for Semiconductors and Semiconductor Devices, Proceedings SPIE Vol. 794, O. H. Glembocki, F. H. Pollak, and J. J. Soong, eds., SPIE, Bellingham, Washington (1987) pp. 242–251.
Y. Demay, D. Arnoult, J. P. Gailliard, and P. Medina, In situ spectroscopic ellipsometry during molecular-beam epitaxy of cadmium mercury telluride, J. Vac. Sci. Technol. **A5**, 3139 (1987).
M. G. Doss, D. Chandler-Horowitz, J. F. Marchiando, S. Krause, and S. Scraphin, Analysis for the characterization of oxygen implanted silicon (SIMOX) by spectroscopic cllipsometry, Materials Research Society Symposia Proceedings Vol. 209, Materials Research Society, Pittsburgh, Pennsylvania (1991) pp. 493–498.
B Drevillon, In situ analysis of the growth of semiconductor materials by phase modulated ellipsometry from UV to IR, in Surface and Interface Analysis of Microelectronic Materials Processing and Growth, Proceedings SPIE Vol. 1186, L. J. Brillson and F. H. Pollak, eds., SPIE, Bellingham, Washington (1989) pp. 110–121.
P. Dutta, G. A. Candela, D. Chandler-Horowitz, and J. F. Marchiando, Nondestructive characterization of oxygen-ion-implanted siliconon insulaotr using multiple-angle ellipsometry, J. Appl. Phys. **64**, 2754–2756 (1988).
K. G. Merkel, P. G. Snyder, J. A. Woollam, and S. A. Alectovitz, GaAs/AIGaAs superlattic characterization by variable angle spectroscopic ellipsometry, in Spectroscopic Characterization Techniques for Semiconductor Technology III, Proccedings SPIE Vol. 946, O. J. Glembocki, F. II. Pollak, and F. Ponce, eds., SPIE, Bellingham, Washington (1988) pp. 105–111.
P. G. Snyder, J. A. Woollam, and S. A. Alterovitz, Variable angle of incidence spectroscopic ellipsometric study of semiconductor multilayer structures, in Materials Characterization, Materials Research Society Symposia Proceedings Vol. 69, N. Cheung and M. A. Nicolet, eds., Materials Research Society, Pittsburgh, Pennsylvania (1986) pp. 245–250.
P. G. Snyder, K. G. Merkel, and J. A. Woollam, Optical measurement of built-in and applied electric fields in AIGaAs/GaAs heterostructures, in Spectroscopic Characterization Techniques for Semiconductor Technology III, Proceedings SPIE Vol. 946, O. J. Glembocki, F. H. Pollak, and F. Ponce, eds., SPIE, Bellingham, Washington (1988) pp. 98–104.
E. Taft and L. Cordes, Optical evidence for a silicon-silicon oxide interlayer, J. Electrochem. Soc. **126**, 131–134 (1979).
J. A. Woollam and P. G. Snyder, Fundamentals and applications of variable angle spectroscopic cllipsometry, Materials Sci. Eng. **B5**, 279–283 (1990).

## 3. Infrared Spectroscopy

### 3.1 Introduction

Infrared (IR) spectroscopy in the range from 10 cm^−1^ to 10,000 cm^−1^ can be used to determine impurity type and concentration in semiconductor materials, film thickness, semiconductor alloy composition, carrier density and scattering time. These determinations can be made for bulk, film, and microstructure systems. One application in Si measures the amount of interstitial oxygen, whose concentration is critical; correct values provide gettering action, reducing the level of other impurities and hence, producing material with low leakage currents. Concentrations of oxygen in silicon and other impurities can be determined by infrared spectroscopic evaluation during processing.

### 3.2 Physical Basis

Infrared radiation interacts with semiconductor lattices, carriers, and impurities, and is affected by semiconductor layer thickness. Binary semiconductors like GaAs have vibrational lattice transverse optical (TO) modes which couple to infrared radiation, with resonant absorption when the incoming frequency matches the TO frequency. Ternary alloys like Al*_x_*Ga_1−_*_x_* As display two TO modes, whose strength and frequency vary with *x*.

Semiconductor impurities can absorb infrared energy by photoionization of their bound carriers or may modify their immediate lattice environment to produce a so-called local vibrational mode (LVM). in the case of photoionization, the impurity must be in a populated or ground state; hence this absorption process is normally observed at cryogenic temperatures. Local vibrational modes occur when an impurity atom is lighter than the host lattice. Impurities important to semiconductor processing such as oxygen and carbon in Si produce LVM absorptions in the infrared region. If a semiconductor film is not too highly absorbing (device grade material is often highly conductive, and, therefore, absorbing), interference between infrared radiation reflected from the front surface, and that reflected from the back, can produce fringes whose spacing is related to the film thickness. Finally, free charge carriers in a semiconductor also absorb electromagnetic radiation. The absorption increases with wavelength; hence, absorption can be significant at infrared wavelengths even for low carrier concentrations.

An important feature of optical processes such as those occurring in the IR region is that quantitative measurements can be made based on absorption, reflection, or transmission data, and then accurately described by simple theory. The infrared properties are specified by the complex dielectric function ∊(*ω*) = ∊_1_(*ω*)+i∊_2_(*ω*), which is related to the complex refractive index *ñ*(*ω*)*= n*(*ω*)*+ik*(*ω*)by

$$\begin{array}{c} n^{2}-k^{2}=\epsilon_{1}\left(\omega \right)\\ 2nk=\epsilon_{2}\left(\omega \right). \end{array}$$ (4)

If *ñ*(*ω*) is known, then the reflection and transmission properties can be calculated. For instance, a semiconductor film has at normal incidence a front-surface reflection coefficient *R*,

$$R=\frac{\left[{\left(n-1\right)}^{2}+k^{2}\right]}{\left[{\left(n+1\right)}^{2}+k^{2}\right]}$$ (5)

and a transmission coefficient,

$$T=\frac{{\left(1-R\right)}^{2}e^{-\alpha d}}{\left(1-R^{2}e^{-2\alpha d}\right)},$$ (6)

where *α* is the absorption coefficient (= 4π*k*/λ) and *d* is the film thickness. These expressions apply only when interference effects can be neglected, i.e., when noncoherent light is used.

For absorption due to lattice vibrations, or due to local impurity vibrational modes, the dielectric function *ϵ* is

$$\epsilon \left(\omega \right)=\epsilon_{\infty }+\frac{S\omega_{R}^{2}}{\omega_{R}^{2}-\omega^{2}-i\omega \Gamma }.$$ (7)

In this well-known Lorentzian form, ∊*_x_* is the high-frequency limit of *∊*(*ω*); *S* is the oscillator strength; *Γ*is a damping term; and the resonant frequency *ω*_R_is the TO frequency for a lattice oscillation, or characteristic fingerprint frequency for an impurity vibrational mode. For a ternary semiconductor like Al_1−_*_x_*Ga*_x_* As, each TO mode is represented by a resonant term like that in Eq. (7), whose parameters depend on *x.*

If there are free carriers present, *∊*has an additional term $-\epsilon_{\infty }\omega_{p}^{2}/\left[\omega \left(\omega -i/\tau \right)\right]$, where τ is the carrier scattering time, and $\omega_{p}^{2}$ is the plasma frequency 4πNe^2^/m^*^ϵ_∞_, with *N* the carrier concentration and m^*^ the carrier effective mass. Hence, *N* and the drift mobility μ = eτ/m^*^ can be found from these parameters if m^*^ is known. Also, the dc resistivity $\rho =m^{*}/ne^{2}\tau =\omega_{p}^{2}\tau$ can be found from these quantities, even if m^*^ is not known.

From the theory discussed above, measured reflection, transmission, and absorption data can be related to the microscopic semiconductor parameters. Thus, concentrations of impurity oxygen and carbon in silicon, for instance, in the parts-per-million range can be determined. Infrared analysis can also be used to determine carrier concentrations, mobilities, and resistivities for carrier concentrations as low as 10^14^ cm^−3^, with results that agree well with conventional Hall effect and resistivity data.

Further, analysis of infrared reflectivity for thin films of semiconductors, which show interference effects, can be used to accurately determine the thicknesses of films in the micrometer range. For nonabsorbing films, the peaks of observed interference fringes occur at the wavelengths

$$\lambda_{P}=\frac{2n\left(\omega \right)d}{p}$$ (8)

Where *d* is the layer thickness, *n* (*ω*)is the real part of the refractive index, and *p* is the interference order, an integer or half integer 1/2, 1, 3/2…. π (*ω*) is known for semiconductors of interest, so that *d* can be derived from Eq. (8).

Infrared methods can also be used to determine the presence of shallow impurities. A shallow donor impurity behaves like a hydrogen atom immersed in a medium with dielectric constant ∊and conduction band effective mass ratio m^*^/m_0_, where *m*_0_ is the free electron mass. From the Bohr model, the ionization energy (in eV) is

$$E_{\text{ion}}=\frac{13.6}{\epsilon^{2}}\left(\frac{m^{*}}{m_{0}}\right)$$ (9)

which is approximately 6 meV for GaAs. This simple model cannot predict ionization energies for different impurities in different materials, but shows that shallow donor ionization energies lie in the infrared region. Their exact values, and lience identification of the particular impurity, can be found from infrared photoconductivity spectra.

The theory developed above can be used to analyze inhomogeneous microstructures composed of layers of different semiconductors. Each layer is described by the same infrared theory and parameters that define its bulk behavior, to give its complex refractive index. Then, using standard theory for the reflection and transmission at each interface, the total infrared response of the structure can be calculated by computer. This model works well in fitting such data to determine average carrier properties, layer thicknesses, and phonon behavior which is related to microstructurc properties and quality.

### 3.3 Experimental and Technical Details

Infrared spectroscopy often requires only minimal sample preparation, and the low energy and power of infrared radiation sources guarantee that the samples arc not altered by the measurement. Because infrared light typically penetrates several micrometers into a semiconductor, this radiation can also be used to examine the various layered regions of an entire microstructure such as a super-lattice.

Low source intensity and low detector sensitivity in the infrared region make Fourier transform spectroscopy the method of choice for obtaining 1R spectra. In the Fourier method, infrared light, having traversed or been reflected from a sample, is analyzed with an interferometer. The optical intensity reaching the detector through the interferometer is the optical Fourier transform of the desired trans-mission or reflection spectrum. The interference spectrum is computationally transformed back into transformalgorithmona computer. Thelight throughput advantage of a large interferometer aperture rather than the narrow slit of a conventional dispersive monochromator is referred to as the Jacquinot advantage. In addition, the interferometer allows simultaneous observation of many wavelengths, the so-called Fellgett advantage.

**Fig. 3:**
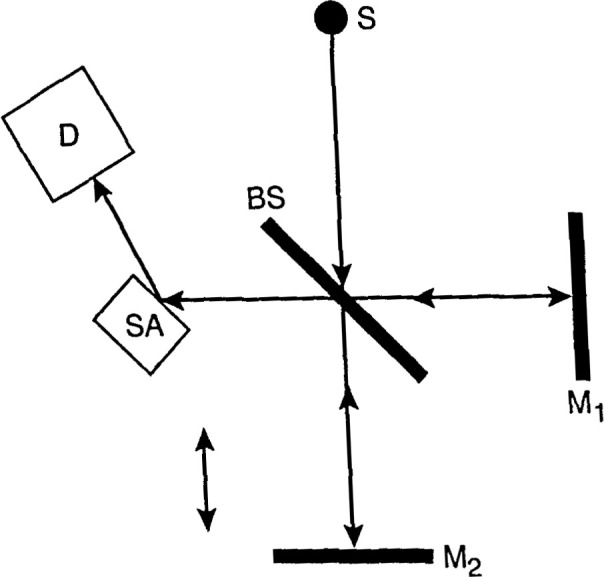
Diagram of a Michctson interferometer configured for sample reflectance measurements. S, souree; BS, beamsplitter; M1, fixed mirror; M2, movable mirror, which moves as indicated by the double-headed arrow; SA, sample; D, detector. The type of source and beamsplitter depends on the region of the infrared where data are to be obtained. The light beams reflected from M1 and M2 recombine to form the interferogram signal which is measured by the detector.

Figure 3 is a schematic diagram of a Michelson interferometer. Radiation fromabroad-band source, *s*, is divided by a beamsplitter, BS. Light reflected from the beamsplitter is also reflectedfrom fixed mirror M1, whereas light transmitted through the beamsplitter is reflected from a movable second mirror, M2. The two light beams re-combine to produce a net intensity whose magnitude depends on the difference *⊿* between the paths that the two beams traverse. As mirror M2 moves, *⊿* varies continuously. The intensity function *I*(*⊿*), called the interferogram, is

$$I\left(\Delta \right)=\int_{0}^{\infty }S\left(f\right)\left[1+\cos\left(2\pi f\Delta \;\right)\right]df,$$ (10)

Where *S*(*f*) is the intensity spectrum of the source as modified by the sample, *and f = ω/πc* is the optical frequency in cm^−1^. Equation (10) is the cosine Fourier transform of *S*(*f*), which can be calculated from the inverse transform

$$S\left(f\right)=\int_{0}^{\infty }\left[I\left(\Delta \right)-\frac{1}{2}I\left(0\right)\right]\cos\left(2\pi f\Delta \;\right)d\Delta .$$ (11)

This is implemented in the laboratory by processing the measured *I*(*⊿*)with a computer to carry out the inverse transform. The spectral resolution in wavenumbers of the Fourier system is 1/*L*, where *L*is the total travel of the movable mirror. Most machines use a rapid scan method, where the mirror is swept through its entire travel in a short time. Many sweeps are averaged together to enhance the signal-to-noise ratio.

Because of the small intensities of infrared sources, especially at the very long wavelengths of the far infrared spectrum, high-quality detectors are important. Liquid helium bolometers give the highest sensitivity, but are expensive and complex to operate. Mercury-cadmium-telluride detectors operating at liquid nitrogen temperatures work well in the mid infrared spectrum. Pyroelectric detectors operate at room temperature and are simple and rugged. They are sufficiently sensitive, from ultraviolet to millimeter wavelengths, for much semiconductor work.

Commercial Fourier transform infrared systems are available that cover the near infrared to the far infrared spectrum, by suitable choice of light source, beam splitter, and detector. To avoid the effect of water vapor absorption on the desired spectrum, these spectrometers are evacuated. Often, semiconductor samples must be cooled in order to better study electronic properties by removing the effects of lattice vibrations or phonons in the absorption spectra. This can be accomplished to 77 K with liquid nitrogen, and to 4.2 K with a liquid helium cryogenic system or by a mechanical refrigerator.

### 3.4 Illustrative Applications

**Fig. 4:**
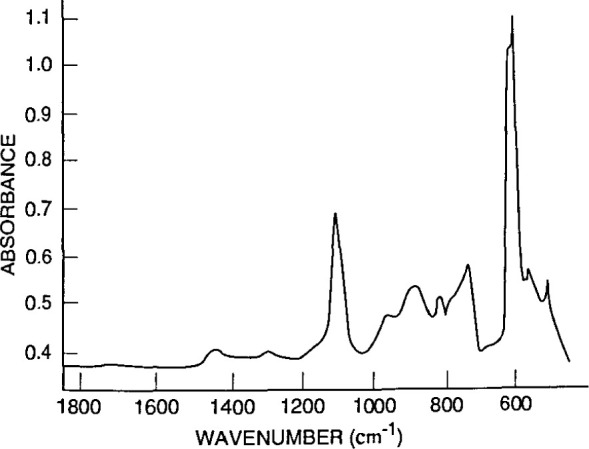
Infrared absorbance for a Czochralski-grown silicon wafer 2 mm thick, derived from transmission spectra using a commercial Fourier spectrometer with a mercury-cadmium-telluride detector. The characteristic interstitial oxygen line at 1107 cm^−1^ and the substitutional carbon line at 605 cm^−1^ appear. Much of the remaining structure is due to silicon phonon modes. The absorbance at 1107 cm^−1^ is linearly related to the oxygen concentration. Calibration data exist to convert absorbance into oxygen concentration in parts per million atomic or atoms per cubic centimeter. (See Sec. 3.5, Applications Refs., Krishnan, Stout, and Watanabe, in Practical Fourier Transform Infrared Spectroscopy, J. R. Ferraro and K. Krishnan, Eds., Academic Press, San Diego (1990), fig. 5, p. 298.)

**Fig. 5:**
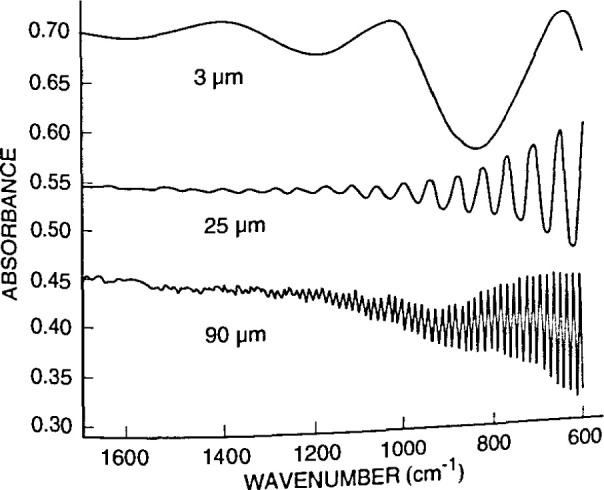
Infrared reflectance spectra from structures consisting of low carrier concentration silicon epitaxial layers onhigh concentration silicon substrates, showing interference fringes that can be used to determine epitaxial layer thickness. Layers of different thickness produce different fringe spaeings, according to Eq. (8). (See Sec. 3.5, Applications Refs., Krishnan, Stout, and Watanabe, in Practical Fourier Transform Infrated Spectroscopy, J. R. Ferraro and K. Krishnan, Eds., Academic Press, San Diego (1990), fig. 25, p. 333.)

**Fig. 6:**
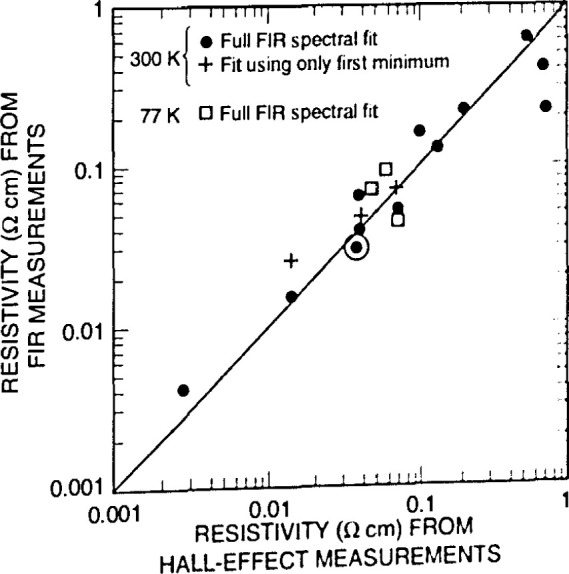
Infrared values for de resistivity compared to resistivity values from standard electrical (resistivity-Hall effect) measurements, for several n- and p-type Hg_1−g_Cd, Te films on CdTc substrates. The films were typically several micrometers thick with *x* values of 0.2 to 0.4. Results at both 300 k and 77 k are shown. Resistivity values marked “Fit using only first minimum” were derived from the measured position of one particular feature of the FIR spectra, the so-called Plasmon-phonon minimum whose location depends on resistivity. The solid line represents perfect agreement between the infrared and the conventional electrical results. The contactless infrared method agrees well with the time-consuming and destructive electrical method requiring contracts. (See See. 3.5, Applications Refs. Jones, Boyd, Konkel, Perkowitz, and Brandstein (1986), fig. 2, p. 2057.)

**Fig. 7:**
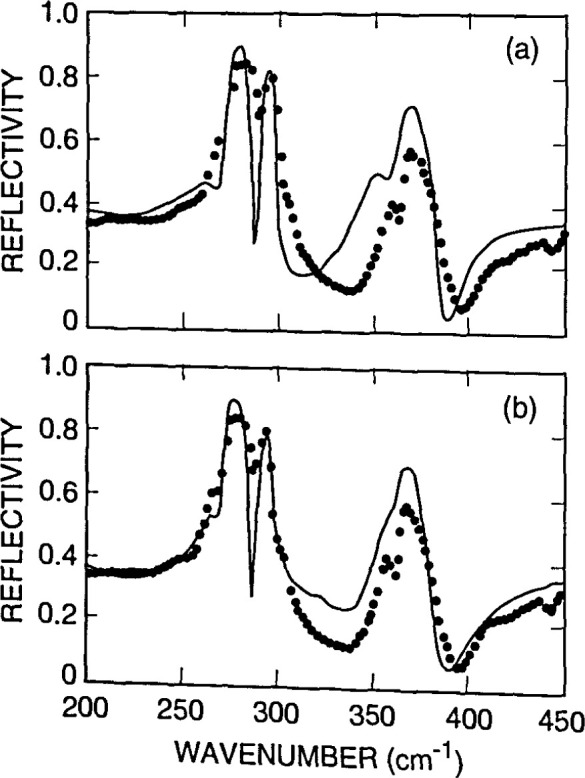
Infrared reflectivity spectra for an AlAs-GaAs superlattice with 50 layer pairs. Panels (a) and (b) show the same data points. The peaks at 275 cm^−1^ and 365 cm^−1^ are the GaAs TO mode and AlAs TO mode, respectively. The peak at 290 cm^−1^ and shoulder at 355 cm^−1^ are interference fringes. The minima inthe spectra lie at the positions of the structure’s longitudinal optical phonon modes, which are sensitive to layer thickness. In Panel (a), the fitted solid line uses known phonon parameters for GaAs and AlAs, and the grower’s nominal layer thickness *d*_AIAs_*=d*_GaAs_=10 nm. The improved fit in Panel (b) uses the same phonon parameters, but allowed each layer thickness to et al. gave *d*_AIAs_ = (7.5 ± 0.2 nm and *d*_GaAs_ = (8 2 ± 0.2) nm, in agreement with the x-ray results *d*_AIAs_= (8.3 *±*0.8) nm, in *d*_G__a__A__s_ = (8.3 ± 0.8)nm.(SeeSec3.5, Applications Refs., Sudharsanan, Perkowitz, Lou, Drummond, and Doyle (1988), fig. 1, p. 658.)

Figure 4 illustrates the absorption peaks for interstitial oxygen at 1107 cm^−1^ and substitutional carbon at 605 cm^−1^ in Czochralski-grown silicon. Such absorption data can be converted into oxygen concentration values, giving a rapid, nondestructive way to determine this important quantity. Figure 5 demonstrates how a semiconductor film, in this case an epitaxial layer of high-resistivity silicon deposited on low-resistivity silicon, gives clear interference fringes that can be used to measure the layer thickness. Figure 6 correlates resistivity obtained from infrared measurements with resistivity obtained from carrier transport measurements. The data, from epitaxial n- and p-type Hg*_x_*Cd_1−_*_x_*Te films, are compared to results from conventional electrical measurements, which require ohmic contacts that can be difficult to apply. Figure 7 shows infrared reflectance data for an AlAs-GaAs superlattice. As the caption discusses in detail, the TO phonon mode for each constituent material is clear, as are interference fringes and other features. The simple theory for infrared phonon response gives a fit which reproduces all the main features of the spectra, and allows an estimale of layer thickness.

Table 4 gives the sensitivities of typical quantities measured by infrared spectroscopy such as interstitial oxygen concentrations in Si and GaAs, substitutional carbon concentrations in Si and GaAs, and B, P, and As concentrations in Si. For more specific details, the reader should refer to the citations given in the table.

### 3.5 References

#### General

R. J. Bell, Introductory Fourier Transform Spectroscopy, Academic Press, New York (1972).
G. L. Carr, S. Perkowitz, and D. B. Tanner, Fat infrated properties of inhomogenus materials, in Infrated and Millimeter Waves, Vol. 13, K. J. Button, ed., Academic Press, New York (1985) pp. 171–263.
E. D. Palik and R. T. Holm, Optical characterization of semiconductors, in Nondestructive Evaluation of Semiconductor Materials and Devices, J. N. Zemcl, ed., Plenum, New York (1979) pp. 315–345.
S. Perkowitz, Submillimeter solid state physics, in Infrared and Millimeter Waves, Vol. 8, K. J. Button, ed., Academic Press, New York (1983) pp. 71–125.

#### Applications

P. M. Amirtharaj. G. Holah, and S. Perkowitz, Far infrared spectroscopic study of In_1−_*_x_*Ga*_x_*As_y_P_1−y_, Phys. Rev. **B 21**, 5656–5661 (1980).
G. J. Brown and W. C. Mitchel, Mid-infrared spectral response of semi-insulating GaAs, in Impurities, Defects and Diffusion in Semiconductors: Bulk and Layered Structures, Materials Research Society Symposia Proceedings Vol. 163, D. J. Wolford, J. Bernholc, and E. E. Haller, eds., Materials Research Society, Pittsburgh, Pennsylvania (1989) pp. 157–162.
J. P. Fillard, M. Castagne, J. Bonnafe, and J. Gall, Scattering and absorption of infrared light on EL2 clusters in GaAs semi-insulating materials, in Materials Characterization, Materials Research Society Symposia Proceedings Vol. 69, N. Cheung and M.-A. Nicolet, cds., Materials Research Society, Pittsburgh, Pennsylvania (1986) pp. 231–236.
D. K. Gaskill, J. Davis, R. S. Sillmon, and M. N. Sydor, Nondestructive characterization of carrier concentration and thickness uniformity for semiconductors using infrared reflectance spectroscopy, in Modern Optical Characterization Techniques for Semiconductors and Semiconductor Devices, Proceedings SPIE Vol. 794, O. H. Glembocki, F. H. Pollak, and J. J. Soong, eds., SPIE, Bellingham, Washington (1987) pp. 231–241.
J. Geist, Infrared absorption cross section of arsenic in silicon in the impurity band region of concentration, Appl. Optics **28**, 1193–1199(1988).
C. E. Jones, T. N. Casselman, J. P. Faurie, S. Perkowitz, and J. Schulman, Infrared properties and bandgaps of HgTc/CdTe superlattices, Appl. Phys. Lett. **47**, 140–142 (1985).
C. E. Jones, M. E. Boyd, W. H. Konkel, S. Perkowitz, and R. Braunstein, Noncontact electrical characterization of epitaxial HgCdTe, J. Vac. Sci. Technol. **A4**, 2056–2060 (1986).
K. Krishnan, Precise and Rapid Measurement of Interstitial Oxygen Concentration in Silieon, Bio-Rad Semiconductor Notes No. 102, Bio-Rad Semiconductor Measurement Systems, 237 Putnam Ave., Cambridge, MA 02139, April 1983.
K. Krishnan, A study of the spatial distribution of the oxygen content in silicon wafers using an infrared transmission microscope, Bio-Rad Semiconductor Notes No. 105, Bio-Rad Semiconductor Measurement Systems, 237 Putnam Ave., Cambridge, MA 02139, January 1985.
K. Krishnan and R. B. Mundhe, Characterization of semiconducting silicon using FT-IR spectroscopy, in Spectroscopic Characterization Techniques for Semiconductor Technology, Proceedings SPIE Vol. 452, F. H. Pollak and R. S. Bauer, eds., SPIE, Bellingham, Washington (1983) pp. 71–78.
K. Krishnan, P. J. Stout, and M. Watanabe, Characterization of semiconductor silicon using Fourier transform infrared spectrometry, in Practical Fourier Transform Infrared Spectroscopy, J. R. Ferraro and K. Krishnan, eds., Academic Press, San Diego (1990) pp. 285–349.
B. Lou, S. Perkowitz, and R. Sudharsanan, Anisotropy and infrared response of the AIAs-GaAs superlattice, Phys. Rev. B 38, 2212–2214 (1988). [Erratum: Phys. Rev. B **39**, 1387 (1989)]
E. Merk, J. Heyman, and E. E. Haller, Infrared absorption study of zinc-doped silicon, in Impurities, Defects and Diffusion in Semiconductors: Bulk and Layered Structures, Volume 163, Materials Research Society Symposia Proceedings, D. J. Wolford, J. Bernholc, and E. E. Haller, eds., Materials Research Society, Pittsburgh, Pennsylvania (1989) pp. 15–20.
W. J. Moore, Infrared transmission characterization of p-type gallium arsenide, in Optical Characterization Techniques for Semiconductor Technology, Proceedings SPIE Vol. 276, D. E. Aspnes, S. So, and R. F. Potter, eds., SPIE, Bellingham, Washington (1981) pp. 101–103.
R. C. Newman, Localized vibrational mode spectroscopy of impurities in semiconductor crystals, in Growth and Characterization of Semiconductors, R.A. Stradling and P.C. Klipstein, eds., Adam Hitger, Bristol (1990) pp. 105–118.
S. Perkowitz and J. Breecher, Characterization of GaAs by far infrared reflectivity, Infrared Phys. **13**, 321–326 (1973).
S. Perkowitz, Far infrared characterization of Hg*_x_*Cd_1−_*_x_*Te and related electronic materials, J. Electronic Materials **14**, 551–562 (1985).
S. Perkowitz, D. Rajavel, I. K. Sou, J. Reno, J. P. Faurie, C. E. Jones, T. Casselman, K. A. Harris, J. W. Cook, and J. F. Schctzina, Far infrared study of alloying in HgTe-CdTe superlattices, Appl. Phys. Lett. **49**, 806–809 (1986).
S. Perkowitz, Far infrared spectroscopy of Hg*_x_*Cd_1−_*_x_*Tc and related materials, in Far-Infrared Science and Technology, Proceedings SPIE Vol. 666, J. R. Izatt, ed., SPIE, Bellingham, Washington (1986) pp. 120–125.
S. Perkowitz, R. Sudharsanan, and S. S. Yom, Far infrared analysis of alloy structure in HgTe-CdTe superlattices, J. Vac. Sci. Technot. **A5**, 3157–3160 (1987).
S. Perkowitz, R. Sudharsanan, S. S. Yom, and T. J. Drummond, AIAs phonon parameters and heterostructure characterization, Solid State Commun. **62**, 645–647 (1987).
B. Senitzky and S. P. Weeks, Infrared reflectance spectra of thin-epitaxial silicon layers, in Optical Characterization Techniques for Semiconductor Technology, Proceedings SPIE Vol. 276, D. E. Aspnes, S. So, and R. F. Potter, eds., SPIE, Bellingham, Washington (1981) pp. 222–226.
R. Sudharsanan, S. Perkowitz, S. S. Yom, and T. J. Drummond, Far infrared reflectance spectroscopy of AlAs-GaAs microstructures, in Modern Optical Characterization Techniques for Semiconductors and Semiconductor Devices, Proceedings SPIE Vol. 794, O. H. Glembocki, F. H. Pollak, and J. J. Soong, eds., SPIE, Bellingham, Washington (1987) pp. 197–201.
R. Sudharsanan, S. Perkowitz, B. Lou, T. J. Drummond, and B. L. Doyle, Far-infrared characterization of AlAs-GaAs superlattice structure, Superlattices and Microstructures **4**, 657–660 (1988).
L. E. Taroff, C. J. Miner, and A. J. Springthorpe, Epitaxial layer thickness measurements by reflection spectroscopy, J. Electronic Materials **18**, 361–367 (1989).
W. M. Theis, C. W. Litton, and K. K. Bajaj. Infrared localized mode spectroscopy of carbon-implanted GaAs, in Optional Characterization Techniques for Semiconductor Technology, Proceedings SPIE Vol. 276, D. E. Aspnes, S. So, and R.F. Potter ed., SPIE, Bellingham, Washington (1981) pp. 109–112.
J. Vindevoghel, M. Vindevoghel, and Y. Leroy, Millimetric and far infrared conductivity for p-Si. Evidence for interband transitions, Infrared Phys. **18**, 99–105 (1978).
J. M. Zavada, H. A. Jenkinson, and T. J. Gavanis, Optical properties of proton implanted n-typc GaAs, in Optical Characterization Techniques for Semiconductor Technology, Proceedings SPIE Vol. 276, D. E. Aspnes, S. So, and R. F. Potter, eds., SptE, Bellingham, Washington (1981) pp. 104–108.

## 4. Optical Microscopy

### 4.1 Introduction

In applications where the dimensions of interest are below the optical diffraction limit (~0.8 μm), electron microscopy is used by necessity. However, traditional optical methods remain useful for a large number of applications such as examining topological features larger than ∼1.0 μm, examining defects, or counting etehpits. Several specialized forms of optical microscopy are highly valuable: Nomarski, scanning laser, and microspectrophotometry. In Nomarski microscopy, interference methods are used to increase the contrast between small differences in the surface level of a semiconductor wafer. Scanning microscopy in both the visible and infrared spectral ranges allows two-dimensional imaging of features in a layer or structure. Finally, microspectrophotometry allows film thickness determination from spectral analysis of reflected light.

Scanning microscopy is also used in both the visible and the infrared spectral ranges to form two-dimensional images of inhomogeneities in a semiconductor. The form called confocal microscopy produces three-dimensional images [1]. One visible light-scanning technique of special interest is the optical-beam-induced current method (abbreviated OBIC, or sometimes LBIC, for laser-beam-induced current), which detects grain boundaries, dislocations, and other defects in semiconductors and semiconductor devices. OBIC images represent spatialdistributions of electrically active defects that include inclusions, strain, damage precipitates, stacking faults, twin boundaries, dislocation clusters, and bandgap and doping variations. In this technique, a focused laser beam is scanned across the surface of the sample, and the induced current between two remote contacts on the sample is measured as a function of the laser beam position. The induced current is a result of the charge-separation effect of the regions in the sample which is homogeneous and defect-free does not generate any induced current. Infrared scanning has been used to study individual precipitate particles in Si ingots, and to examine GaAs and other materials.

### 4.2 Physical Basis

#### 4.2.1 Nomarski Microscopy

In Nomarski microscopy, two microscopic images of a surface are formed so that they are slightly displaced in space and of opposite phase. Interference bands appear where the images overlap. The physical displacement and the interference bands heighten the visibility of small variations in surface levels.

#### 4.2.2 Scanning Microscopy

In scanning microscopy, a spot of light, whose size is limited by diffraction, is scanned over a specimen. The image of the specimen is developed point by point in sequential fashion, to be displayed or stored for analysis. If the specimen is broadly illuminated and scanned in a raster pattern by a point detector (or raster scanned by a point source, with the light sensed by a broad area detector), a two-dimensional image results. In the variation known as confocal scanning, the specimen is illuminated in only a small region at any one time, and a point detector senses light only from that same region. This makes it possible to develop a three-dimensional image. Confocal scanning also enhances resolution.

The light can be sensed by any of several conventional detectors. In the OBIC method, however, the detector is an external circuit that measures the current produced locally by the incident light. Light intensity from a laser of even modest power creates a high density of carriers in the sample, due to electron-hole excitation. The electrons and holes are affected by the electric fields associated with macroscopic defects, such as grain boundaries in polycrystalline silicon, so that the motion of the electrons and holes induces a current which is sensed by an external circuit. Hence, OBIC images clearly show the presence of defects, and map out their locations.

#### 4.2.3 Microspectrophotometry

Reflection spectrophotometry depends on the interference pattern caused by reflections from top and bottom surfaces of a transparent film. The equations governing reflection from stratified dielectric media are derived in most optics texts [2]. Microspectrophotometry is normally used for determining the film thickness of a single layer on a substrate or the film thicknesses in a relatively simple multilayer stack. As in ellipsometry, values of the functions *n*(*ω*)and *k* (*ω*) for each of the layers of interest are needed to determine the thicknesses. The advantages ot reflectometry relative to ellipsometry are that most of the information is carried in the wavelength dependences, and it is relatively simple to focus the beam down to spot sizes on the order of micrometers [3].

### 4.3 Experimental and Technical Details

#### 4.3.1 Nomarski microscopy

**Fig. 8:**
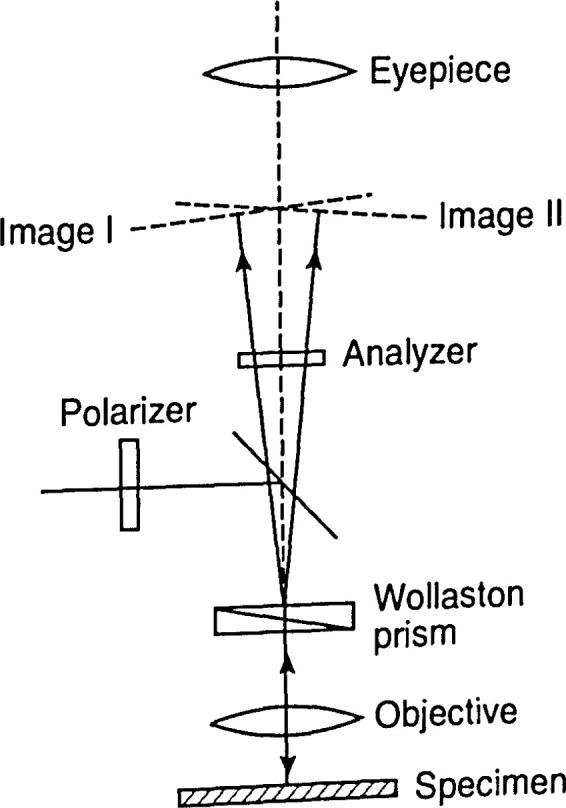
Scheamatic diagram of a Nomarski interference microscope. The Wollaston prism consists of identical prisms of crystalline quartz, one with its optical axis parallel to the plane of the paper and the specimen surface, the other perpendicular to the plane of the paper. (See See. 4.5, General Refs., Modin and Modin (1973), fig. 3.17, p. 130.)

In Nomarski microscopy, two microscopic images of a surface are formed by a Wollaston prism. The prism is placed between the eyepiece and the objective of the microscope, as shown in Fig. 8.

Light traversing the prism is divided into two beams polarized at right angles to each other and diverging by some angle, giving two microscopic images. The microscope includes a polarizer set so that the incident light lies at 45° to the plane of vibration of the prism. With an analyzer set at right angles to the polarizer, this gives two images of the same intensity but 180° apart in phase. Hence, interference bands form where the images overlap. These fringes, combined with the displacement of the images, magnify surface variations. The edges of surface features become clearly visible, and the thickness of films deposited on the surface can be found.

#### 4.3.2 Scanning

**Fig. 9:**
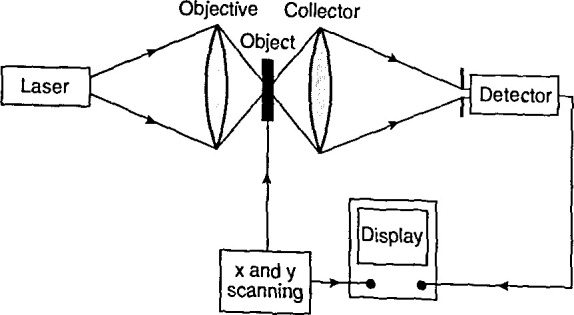
Schematic diagram showing the main elements of a scanning microscope.(See Sec. 4.5, General Refs., Wilson and Sheppard (1984), fig. 1.1, p. 2.)

Microscopy Figure 9 shows the main components of a scanning microscope. It includes a light source, usually a laser; a scanning system, which either moves the laser beam across a fixed sample or moves the sample relative to a fixed optical system; optical elements to focus and manipulate the beam; and a detector. The type of detector used depends on the scanning and imaging methods and on the wavelengths; it may be a single photomultiplier tube or a detector array. In the OBIC method, it is an external circuit that measures the photocurrent.

As stated above, in some systems, the light beam is scanned across a fixed sample. This allows rapid acquisition and display of images; however, there are complications in designing the movable optical system, and in maintaining good image quality. In other designs, the light beam is fixed and the sample is moved to produce the raster pattern. Although these systems are relatively slow, the optical design is simple and produces images of high quality.

**Fig. 10:**
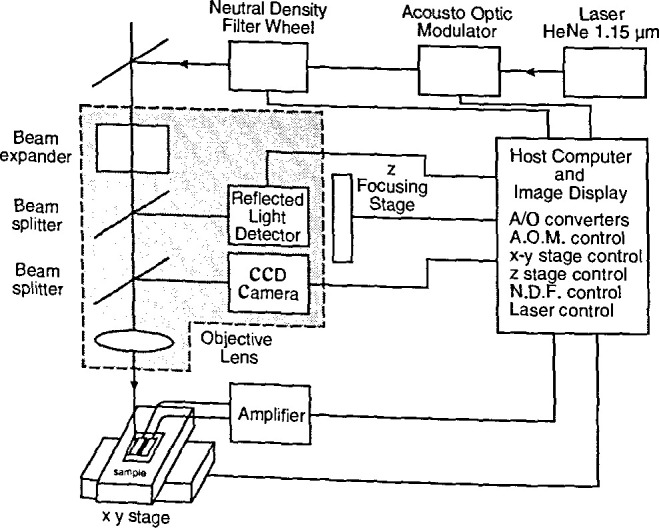
Schematic diagram of an optical-beam-induced current (OBIC) system, also called LBIC (laser-beam-induced current). (See Sec. 4.5, General Refs., Moore, Hennessy, Bajaj, and Tenant (1988), fig. 1.)

The OBIC technique (a typical experimental arrangement is illustrated in Fig. 10) is one of the most important for semiconductor materials and devices, and can readily be implemented with small lasers as sources. A 1 mW HeNe laser produces 3 × 10^15^ photons per second. Based on a calculation using typical parameters for electron-hole generation in a semiconductor, this intensity is enough to generate a large density of electron-hole pairs, about 10^20^ cm^−3^. The electric fields associated with defects or doped regions separate the electron and hole in each pair. These separated carriers can induce a current by flowing through an appropriate external circuit. (Depending on whether the sample includes a p-n junction or not and on the nature of the circuit, either photovoltages or photocurrents can be measured.)

Scanning methods can be used equally well in the visible and in the infrared regions. In one typical infrared system, the source is a semiconductor laser operating at 1.3 μm wavelength (giving a spot diameter of about 2 μm), with detection accomplished by germanium photodiodes. The sample is mechanically moved to produce raster scanning, and the resulting images are taken at resolutions of 512 pixels by 512 pixels.

#### 4.3.3 Microspectrophotometry

Interference of light waves reflected from each interface of a multilayer film structure determines the reflectance of the structure. The reflectance spectrum depends on angle of incidence of the radiation, the refractive indices of the media, polarization of the radiation, and film thicknesses [4]. Whereas the same equations describing reflection and transmission apply in both ellipsometry and reflectance spectrophotometry, the problem is somewhat simplified in the case of reflectometry, where polarization is usually ignored. Normally, the reflected light intensity is recorded versus wavelength. Then, the thicknesses are calculated by fitting measured spectra to calculated spectra based on a model of the layer structure and known dielectric constants. Measurements can also be made of the reflectance versus polarization angle or versus angle of incidence, but this is not normally done in microscopic measurements because these parameters are difficult to change systematically within the microscope environment. The most frequent application of microspectrophotometry is the determination of thicknesses of simple dielectric stacks on a substrate; but microspectrophotometry can also be used like ellipsometry to find the dielectric function of film layers and, hence, film layer composition [4]. Because of the relaxed constraint on the angle of incidence and the relative speed of processing data, microspectrophotometry is an ideal way to map the uniformity of wafer film thickness.

### 4.4 Illustrative Applications

**Fig. 11:**
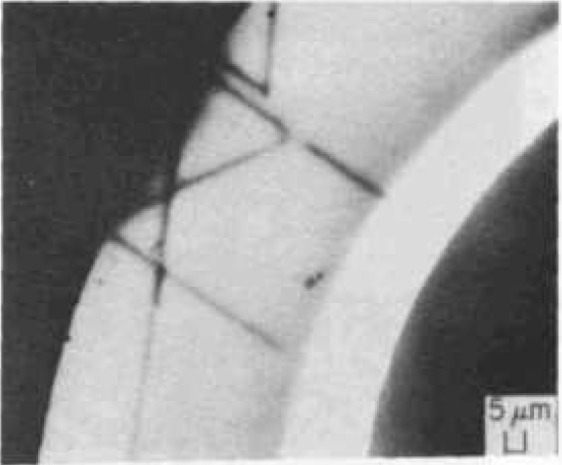
An ODIC image formed by monitoring the emitter-base current in a silicon transistor while a laser beam is scanned across the transistor. The dark straight lines are lines of dislocations in the silicon. (See Sec. 4.5. General Refs., Wilson and Sheppard (1984), fig. 1.6. p. 8.)

**Fig. 12:**
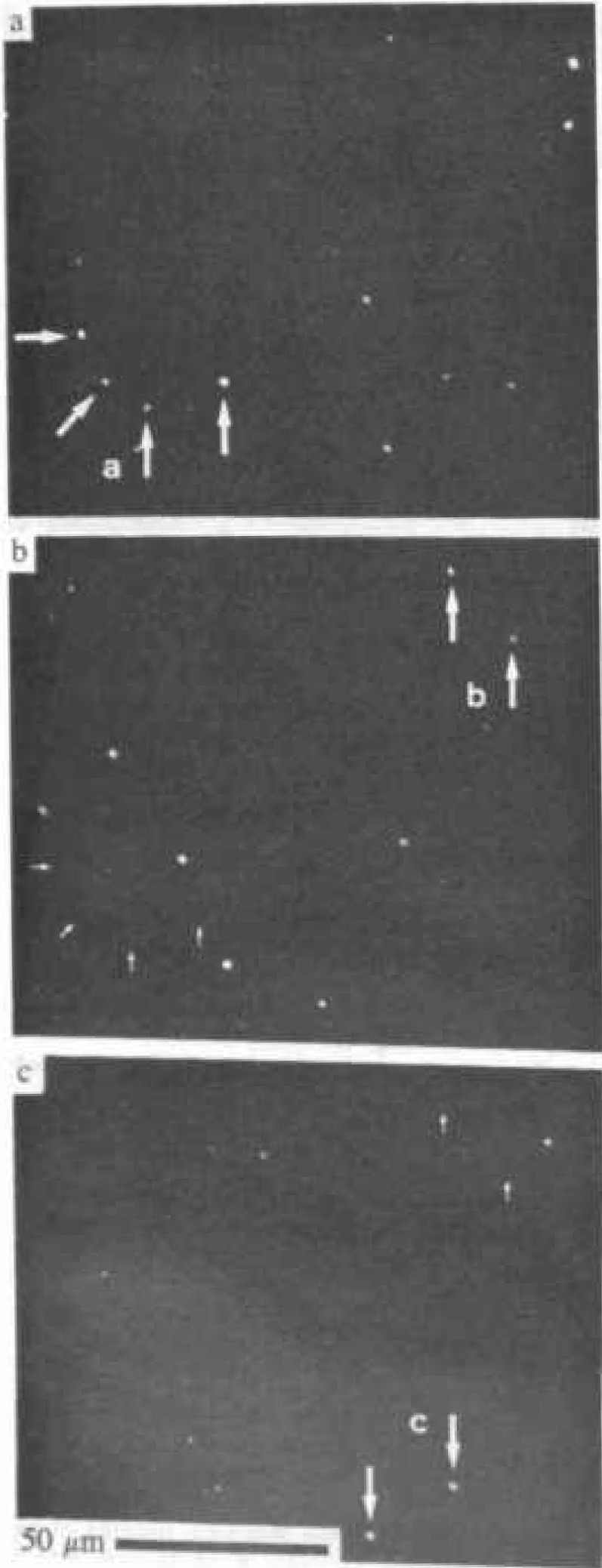
Infrared scanning microscope images of oxide particles inheat-treated(100) Czochralski silicon. The microscope used a semiconductor laser emitting at 1.3 μm, to give a spot size of*−*2 µm. Particles of this size or greater are directly imaged. Smaller particles can still be seen, although as spots 2 µm across, because the system can detect intensity variations of about 0.5 % The depth of focus is 30 µm. Panels (b) and (c) show successively deeper probes into the sample, relative to Pane. (a). The focal plane is 60 µm deeper in Panel (b) and 120 µm deeper in panel (c). The sets of oxide images marked “a,”“b,” and “c” can be followed in and out of focus through the panels. (See Sec. 4.5, Applications Refs., Laczik, Booker, Falster, and Shaw (1989), fig. 1, p. 808.)

Figure 11 shows the power of OBIC imaging to detect flaws in semiconductor materials such as silicon, even when the material is incorporated in an operating device such as a transistor. Figure 12 shows an infrared scanning system micrograph of oxide particles embedded in Czochralski-grown silicon, even displaying those particles smaller than the infrared beam diameter of 2 μm. By focusing to different depths in the sample, it is possible to obtain some depth-dependent information as well.

### 4.5 References

[1] G. O. Ziao, T. R. Corle, and G. S. Kino, Real-time confocal scanning optical microscope, Appl. Phys. Lett. **53**, 716 (1988).
[2] M. Born and E. Wolf, Principles of Optics, Pergamon Press, New York (1975) p. 61.
[3] P. Burggraf, How thick are you thin films?, Semiconductor International (1988) p. 96.
[4] S. E. Stokoeski, Measuring refractive indices of films on semiconductors by microreflectrometry, in Integrated Circuit Metrology, Inspection, and Process Control IV, Proccedings SPIE, W. H. Arnold, ed., SPIE, Bellingham, Washington (1990) p. 253.

#### General

Microscopy of Semiconducting Materials, Institute of Physics Conference Proc. 60, A. G. Cullis and D. C. Joy, eds., Institute of Physics, Adam Hilger, Bristol (1981).
Microscopy of Semiconducting Materials, Institute of Physics Conference Proc. 76, A. G. Cullis, S. M. Davison, and G. R. Booker, eds., Institute of Physics, Adam Hilger, Bristol (1983).
Microscopy of Semiconducting Materials, Institute of Physics Conference Proc. 87, A. G. Cullis and P. D. Augustus, eds., Institute of Physics, Adam Hilger, Bristol (1987).
Microscopy of semiconducting Materials, Institute of Physics Conference Proc. 100, A. G. Cullis and J. L. Hutchinson, eds., Institute of Physics, Adam Hilger, Bristol (1989).
Microscopy of Semiconducting Materials, Institute of Physics Conference Proc. 117, A. G. Cullis and N. J. Long, eds., Institute of Physics, Bristol (1991).
S. Hildebrandt and W. Hergert, Unified theoretical description of the CL, EBIC, PL, and EBIC contrast profile area of an individual surface-parallel dislocation. Phys. Stat. Sol (a) **119**, 689–699 (1990).
R. Keeler, Confocal microscopes, R&D Magazine (April 1991) pp. 40–42.
H. Modin and S. Modin, Metallurgical Microscopy, John Wiley, New York (1973).
T. Wilson and C. Sheppard, Theory and Practice of Scanning Optical Microscopy, Academic Press, London (1984).

#### Applications

J. Bajaj, L. O. Bubulac, P. R. Newman, and W. Tennant, Spatial characterization of semiconductors using laser beam induced current (LBIC), in Modern Optical Characterization Techniques for Semiconductors and Semiconductor Devices, Proceedings SPIE Vol. 794, O. J. Glembocki, F. H. Pollak, and J. J. Song, eds., SPIE, Bellingham, Washington (1987) pp. 136–141.
J. Bajaj, W. E. Tennant, and P. R. Newman, Laser beam induced current imaging of surface nonuniformity at the HgCdTc/ZnS interface. J. Vac. Sci. Technol. A **6**, 2757 (1988).
S. Haq, G. Hobson, K. E. Singer, W. S. Truscott, and J. O. Williams, A transmission electron microscopy investigation of GaAS_1−y_Sb_y−_GaAs superlattices grown by molecular beam epitaxy, in Microscopy of Semiconducting Materials, Institute of Physics Conference Proc. 100, A. G. Cullis and J. L. Hutchinson, cds., Adam Hilger, Bristol (1989) pp. 337–341.
P. Kidd, G. R. Booker, and D. J. Stirland, 3-D distribution of inhomogeneities in LEC GaAs using infra-red laser scanning microscopy, in Microscopy of Semiconducting Materials, Institute of Physics Conference Proc. 87, A. G. Cullis and P. D. Augustus, eds., Adam Hilger, Bristol (1987) pp. 275–280.
Z. Laczik, G. R. Booker, R. Falster, and N. Shaw, Investigation of precipitate particles in Si and CdTc ingot material using the scanning infrared-red microscope (SIRM), in Microscopy of Semiconducting Materials, Institute of Physics Conference Proc. 100, A. G. Cullis and J. L. Hutchinson, cds., Adam Hilger, Bristol (1989) pp. 807–812.
Y-C., Lu, R. K. Route, D. Elwell, and R. S. Feigelson, Etch pit studies in CdTe crystals. J. Vac. Sci. Tcchnol. A **3**, 264 (1985).
J. L. Mariani, B. Pichaud, F. Minari, and S. Martinuzzi, Direct determination of the recombination activity of dislocations in FZ silicon by LBIC measurements, in Microscopy of Semiconducting Materials, Institute of Physics Conference Proc. 100, A. G. Cullis and J. L. Hutchinson, eds., Adam Hilger, Bristol (1989) pp. 801–806.
C. J. L. Moore, J. Hennessy, J. Bajaj, and W. E. Tennant, Finding faults in focal plane arrays, Photonics Spectra (September 1988) pp. 161–166.
M. Ogura, M. Tajima, and Y. Tokumaru, Scanning optical fiber microscope for high resolution laser beam induced current images of semiconductor materials, in Materials Characterization, Materials Research Society Symposia Proceedings Vol. 69, N. Cheung and M.-A. Nieolet, eds., Materials Research Soeiety, Pittsburgh, Pennsylvania (1986) pp. 251–256.
D. J. Stirland, P. Kidd, G. R. Booker, S. Clark, D. T. J. Hurle, M. R. Brozel, and I. Grant, The behaviour of arsenic-rich defects in quenched semi-insulating GaAs, in Microscopy of Semiconducting Materials, Institute of Physics Conference Proc. 100, A. G. Cullis and J. L. Hutchinson, eds., Adam Hilger, Bristol (1989) pp. 373–378.

## 5. Modulation Spectroscopy

### 5.1 Introduction

Modulation spectroscopy is a sensitive technique which can determine fine details of interband transitions in semiconductors. In semiconductor superlattices and other microstructures, detailed knowledge of the complex interband transitions can be used to characterize quantum well widths, potential barrier heights and widths, electric fields, and the amount of strain in strain layer systems.

### 5.2 Physical Basis

The principle behind modulation spectroscopy is that a periodic physical perturbation applied to a sample elicits the derivative of the sample’s optical response to that perturbation. The derivative feature amplifies weak features in the response function and suppresses large constant background levels. This gives modulation methods very high sensitivity to small spectral features that are invisible in conventional spectroscopy.

To illustrate the origin of the derivative response, consider the reflectivity *R* of a sample. This depends on the sample’s dielectric function, which depends on many physical properties. For example, the dielectric function depends on an applied electric field *E*; hence, *R* also depends on *E.* If the applied electric field has a dc componenl *E*_0_ and a small ac component*E*_1_ cos*Ωt* (*Ω* is the modulation angular frequency), the reflectivity can be written as *R*(*E*)*=R*(*E*_o_*+E*_l_cos*Ωt*). If *E*_1_<<*E*_0_, this expression can be expanded in a Taylor series, where only the first two terms are kept; that is

$$R\left(E\right)≅R\left(E_{0}\right)+\frac{dR}{dE}\left(E_{1}\;\cos\;\Omega t\right).$$ (12)

The first term depends on *E*_0_ but not on time, whereas the second term is a periodic function of time at the modulation frequency *Ω.* Hence, the ac portion of the reflectance at frequency *Ω* can be deleted with a lock-in amplifier; this signal is proportional to the derivative d*R*/d*E.* Thus, small structures in the optical spectrum of *R*(*E*) are enhanced, even with the sample at room temperature. A periodic perturbation can be applied to any physical property affecting the sample’s optical response. Examples arc electroreflectance, where a periodic electric field is applied to a sample while its reflectance spectrum is measured; and photoreflectance, where optically injected carriers from a chopped laser beam modulate the “built-in” surface or internal electric fields, thereby modulating the reflectance of the sample. Other forms of modulation spectroscopy have been reviewed by Aspnes [1]. The following discussion concentrates on electroreflectance and photoreflectance, two forms of modulation spectroscopy currently in common usage. Because photoreflectance results from the modulation of “built-in” electric fields, this discussion applies generally to either electroreflectance or photoreflectance.

The enhancement of spectroscopic structures that appear at energies corresponding to energy gaps and other critical points in the joint density of states of the material under study is one useful result of modulation methods. The method becomes more useful still when the measured spectral line shapes can be connected to microscopic parameters through theory. The relationship that makes this connection is [2]

$$\frac{\Delta R}{R}=\alpha \Delta \epsilon_{1}+\beta \Delta \epsilon_{2}$$ (13)

where *⊿R* is the change in reflectivity due to the applied modulation, *⊿*ϵ_1_ and *⊿*ϵ_2_ are the changes in the real and imaginary parts, respectively, of the complex dielectric function ϵ= ϵ_1_+*i*ϵ_2_, and α and *β* are called the Seraphin coefficients. Near the energy gap of a bulk sample, *β*= 0; however, for complex microstructures where interference effects occur, both *α* and *β*need to be considered, and hence *⊿*ϵ_1_ and *⊿*ϵ_2_ must both be known. These can be calculated from general band theory and from dielectric function theory. In the case of electroreflectance and photoreflectance, different spectral line shapes are obtained, and concomittantly different analyses are required, depending on the magnitude of the electric field. The modulation field is usually described in terms of three regimes [2]: high field (Stark effect), intermediate field (Franz-Keldysh effect), or low field. The high field case isnot usually studied under modulation conditions, as a high electric field breaks down the normal selection rules and results in a Stark shift of the band structure. The analysis of spectra based on intermediate and low field theories is discussed in reference to representative spectra, in the illustrative applications section below.

### 5.3 Experimental and Technical Details

**Fig. 13:**
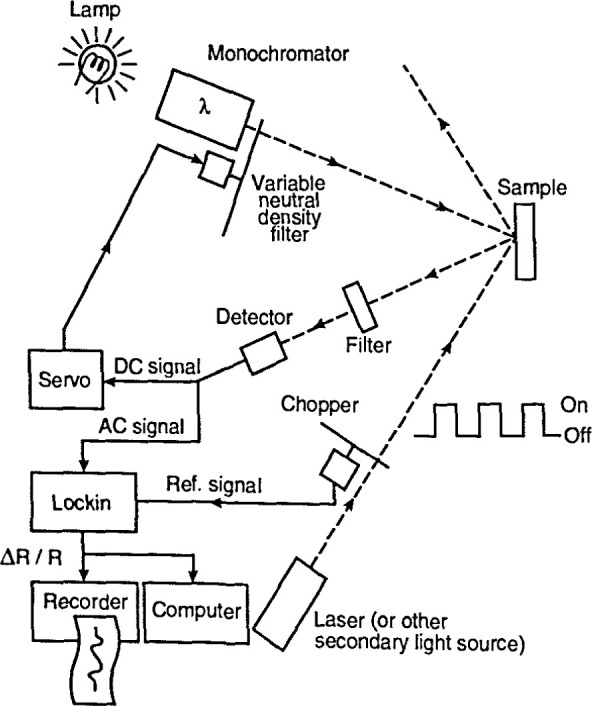
Diagram of a photoreflectance spectrometer, illustrating lamp with following optics and electronics to obtain the spectrum and a laser to supply modulated light. The variable neutral density filter holds the constant part of the detected signal independent of wavelength, facilitating evaluation of the ratio of ∆*R*/*R.* (See Sec. 5.5, General Refs., Pollak and Glembocki (1988), fig. 4, p. 25.)

A modulation measurement requires a light source, a monochromator, and a detector as in conventional spectroscopy, and a means to apply the modulation to the sample. These elements are shown in Fig. 13. The source can be an incandescent or discharge lamp. Generally, a monochromator of 0.25 m focal length provides adequate energy resolution, but higher resolution may be needed in some cases.

The light reflected from the sample is detected by a photomultiplier tube or a photodiode. It contains a steady (dc) component *RI*_0_ (*I*_0_ is the incident light intensity) and a periodically modulated (ac)component *∆RI*_0_. To obtain *∆R/R*, the dc signal and the ac signal must be separately measured and then a ratio of these signals is taken. It is also possible to electronically hold the dc signal (corresponding to *RI*_0_)constant during the measurement. This can be done by a feedback loop that changes the gain of the detector to keep its dc output constant, or, as shown in Fig. 13, by mounting a circular continuously variable neutral density filter on a servo motor and inserting it before the sample.

For photoreflectance, light from a modulated optical source such as a laser, whose photon energy exceeds the sample’s energy gap, impinges on the sample. For electroreflectance of a doped semiconductor, the varying electric field can be applied between an ohmic contact on the sample’s back surface, and a transparent gate electrode on the front of the sample; 5 nm to 10 nm of deposited gold or aluminum is an adequate electrode. A second method is to put the sample in an electrolyte such as a KCl solution, or an acidic solution. The resulting electric field at the sample surface can be changed by varying a voltage applied between the sample and a platinum counter electrode. Photoreflectance and electroreflectance spectroscopy both provide highly detailed spectra even at room temperature, so that sample cooling is usually not needed.

### 5.4 Illustrative Applications

Figure 14a shows the sensitivity of modulation spectroscopy, by comparing the reflectivity spectrum of GaAs in the interband region to the much more detailed *∆R/R* spectrum obtained by electroreflectance. Figure 14a is illustrative of the low field case. Here the spectra near the energy gap can be fitted using [3]

$$\frac{\Delta R}{R}=R\left[e^{i\theta }{\left(\hbar \omega -E_{g}+i\Gamma \right)}^{-m}\right],$$ (14)

where *ℏω*is the photon energy, *E*_g_ is the gap energy, *θ*is the phase factor, *Γ*is the lifetime broadening parameter, and the quantity m takes on the values 2, 2.5, and, 3 for excitonic, three-dimensional, and two-dimensional critical points, respectively. Hence, the critical point type and energy can be determined from fitting this line shape. Aspnes [3] has also developed a “three-point” method for extracting critical point energies which for simple spectra eliminates the need for a full spectrum fit.

Shown in Fig. 14b [4] is the photoreflectance spectra of a moderately n-type doped (~1 × 10^16^ cm^3^) sample of In_0.53_Ga_0.47_As illustrating the intermediate field line shape. This case can be identified by the presence of oscillatory behavior, the so-called Franz-Keldysh oscillations at energies greater than the energy gap of the material. Modulation intensities are usually greater for the high field case than for the low field case. Materials information (electric fields and energy gap values) is typically extracted from Franz-Keldysh oscillations using the Aspnes asymptotic approximation [5]

$$\begin{array}{c} \frac{\Delta R}{R}={\left(\hbar \omega -E_{g}\right)}^{-1}\exp\frac{\Gamma {\left(\hbar \omega -E_{g}\right)}^{1/2}}{{\left(\hbar \Omega \right)}^{3/2}}\\ \times \cos\left[\theta +\frac{2}{3}{\left(\frac{\hbar \omega -E_{g}}{\hbar \Omega }\right)}^{3/2}\right], \end{array}$$ (15)

where *θ*, *Γ*, and *E*_g_are defined above, and *hΩ*is the electro-optic parameter [*e*^2^*E*^2^*h*^2^/32*π*^2^*μ*]^1/1^. Here, *e* is the electron charge, *E* is the dc electric field, *h* is Planck’s constant, and *μ*is the reduced interband effective mass. Since successive extrema represent a change of *π* in the argument of the cosine term in Eq. 15, the energy of the *j*th extrema [5] can be written as

$$j\pi =\theta +\frac{2}{3}{\left[\frac{{\left(\hbar \omega -E_{g}\right)}_{l}}{\hbar \Omega }\right]}^{3/2}.$$ (16)

Hence the phase factor and electro-optic energy can be obtained from the intercept and slope of a plot of ${\left(h\omega -E_{g}\right)}_{j}^{3/2}$ vs *j*. It is noteworthy that the electric field in a structure under study can be extracted from the electro-optic energy, requiring only independent knowledge of the effective mass of the material. Hence, electroreflectance and photoreflectance provide very important methods for nondestructive determination of surface and junction electric fields. These fields can, in turn, be related to doping densities in a space charge region [6] through the Schottky equation.

**Fig. 15:**
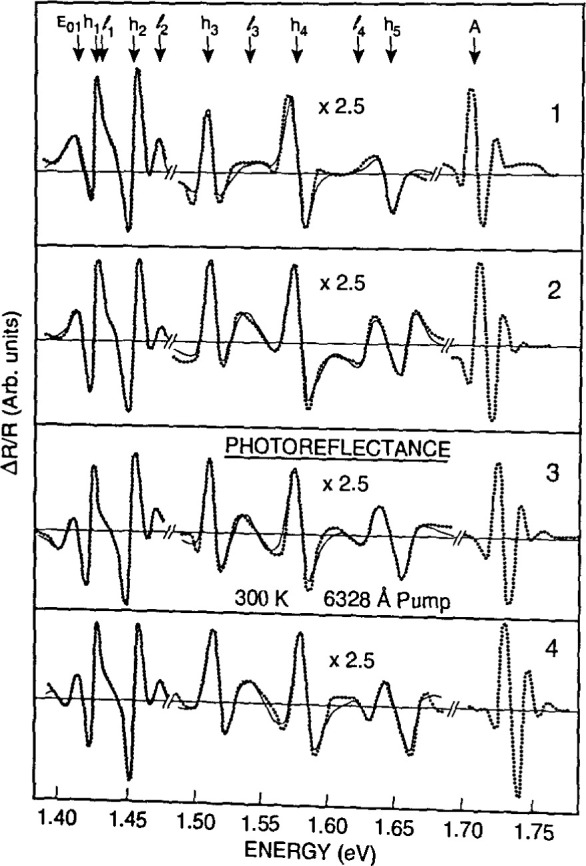
Photoreflectancc spectra of GaAs/Al*_x_*Ga_1−_*_x_*As multiple quantum well (MQW) with nominal *x* value 0.24, and nominal barrier and well thickness of 15 nm and 22 nm, respectively. Spectra (1) to (4) were measured at locations spaced 0.47 cm apart along a straight line. In each, the peak at 1.42 eV marked *E*_o1_ comes from the direct gap of the GaAs substrate, the peak marked “A” near 1.72 eV comes from the direct gap of the Al*x*Ga_1−_*_x_*As barriers, and the remaining features marked *l_h_* and *h_n_*(*n* = 1,2, 3 …) between 1.43 and 1.68 eV come from light and heavy hole interband transitions characteristic of the MQW energy bands. The Al*x*Ga_1−_*_x_*As, *h*_1_, and *l*_1_ features shift with spatial position. Fits to the data show that the well width rages from 21.4 nm to 21.0 nm, and *x* ranges from 0.225 to 0.247, between positions (1) and (4), 1.4 cm apart. (See Sec. 5.5, Applications Refs., Parayanthal, Shen, Pollak, Glembocki, Shanabrook, and Beard (1986), fig. 1, p. 1261.)

Figure 15 presents photoreflectance data used to characterize a GaAs/Al_0.24_Ga_0.76_As multiple quantum well. The chopped laser beam, 100 μm in diameter, had been moved along the structure to give spectra from different spatial regions. Each of theresulting spectra was theoretically fitted, to determine how the well width and Al mole fraction value changed with position over a distance of 1.4 cm.

Table 5 presents the sensitivities to typical quantities measured by photoreflectance spectroscopy such as composition, stress, electric field strength, surface photovoltage, and doping density. For more specific details, the reader should refer to the citations given in the table.

### 5.5 References

[1] D. E. Aspnes, Modulation spectroscopy/elcctric field effects on the dielectric function of semiconductors in Hand book on Semiconductors, Vol.2, T. S. Moss and M. Balkanski, ed., North-Holland, New York (1980) p. 109.
[2] B. O. Seraphin and N. Bottka, Franz-Keldysh effect of the refractive index in semiconductors, Phys. Rev. **139** A560 (1965).
[3] D. E. Aspnes, Third-derivative modulation spectroscopy with low-field elcctroreflectance, Surface Science **37**, 418 (1973).
[4] J. P. Estrera, W. M. Duncan, Y. C. Kao, H. Y. Liu, and E. A. Beam, Systematic optical and x-ray study of In_x_Ga_1−x_As on InP, J. Electronic Materials **20**, 983 (1991).
[5] D. E. Aspnes and A. A. Studna, Schottky-barrier electroreflectance: application to GaAs, Phys. Rev. B **7**, 4605 (1973).
[6] W. M. Duncan and A. F. Schreiner, Surface state study of ion implanted GaAs (Se) from photoreflectance, Solid State Communications **31**, 457 (1979).

#### General

J. I. Pankove, Optical Processes in Semiconductors, Prentice Hall, Englewood Cliffs (1971) pp. 391–407.
F. H. Pollak and O. J. Glembocki, Modulation spectroscopy of semiconductor microstructures: an overview, in Spectroscopic Characterization Techniques for Semiconductor Technology III, Proceedings SPIE Vol. 946, O. J. Glembocki, F. H. Pollak, and F. Ponce, eds., SPIE, Bellingham, Washington (1988) pp. 2–35.

#### Applications

P. M. Amirtharaj, J. H. Dinan, J.J. Kennedy, P. R. Boyd, and O. J. Glembocki, Photoreflectance study of Hg_0.7_Cd_0.3_Te and Cd*_x_*Zn_1−_*_x_*Te E_1_ transition, J. Vac. Sci. Technol. A **4**, 2028 (1986).
R. N. Bhattacharya, H. Shcn, P. Parayanthal, and F. H. Pollak, Elcctrorcflectance and photoreflectance characterization of the space charge region in semiconductors: ITO/InP as a model system, in Modern Optical Characterization Techniques for Semiconductors and Semiconductor Devices, Proceedings SPIE Vol. 794, O. H. Glembocki, F. H. Pollak, and J. J. Soong, eds., SPIE, Bellingham, Washington (1987) pp. 81–87.
R. C. Bowman, R. L. Alt, and K. W. Brown, Photoreflectance spectroscopy studies of alloy composition and ion implant damage in zincbIcnde-type semiconductors, in Modern Optical Characterization Techniques for Semiconductors and Semiconductor Devices, Proceedings SPIE Vol. 794, O. H. Glembocki, F. H. Pollak, and J. J. Soong, eds., SPIE, Bellingham, Washington (1987) pp. 96–104.
R. C. Bowman, D. N. Jamieson, and P. M. Adamson, Optical and structural characterization of boron implanted GaAs, in Spectroscopic Characterization Techniques for Semiconductor Technology III, Proceedings SPIE Vol. 946, O. J. Glembocki, F. H. Pollak, and F. Ponce, eds., SPIE, Bellingham, Washington (1988) pp. 65–75.
J. P. Estrera, W. M. Duncan, Y. C. Kao, H. Y. Liu, and E. A. Beam, Systematic optical and x-ray study of In*_x_*Ga_1−_*_x_*As on InP, J. Electronic Materials **20**, 983–987 (1991).
O. J. Glembocki, Ellipsometric-electrolyte electro-reflectance study of the Si/SiO_2_ interface, in Spectroscopic Characterization Techniques for Semiconductor Technology, Proceedings SPIE Vol. 452, F. H. Pollak and R. S. Bauer, eds., SPIE, Bellingham, Washington (1983) pp. 130–141.
O. J. Glembocki and B. V. Shanabrook, Photoreflectance characterization of microstructures using a dye laser system, in Modern Optical Characterization Techniques for Semiconductors and Semiconductor Devices, Proceedings SPIE Vol. 794, O. H. Glembocki, F. H. Pollak, and J. J. Soong, eds., SPIE Bellingham, Washington (1987) pp. 74–80.
R. Glosser and N. Bottka, Comparative response of electroreflectance and photoreflectance in GaAs, in Modern Optical Characterization Techniques for Semiconductors and Semiconductor Devices, Proceedings SPIE Vol. 794, O. H. Glembocki, F. H. Pollak, and J. J. Soong, eds., SPIE, Bellingham, Washington (1987) pp. 88–95.
T. K. Gupta, Effective bandgap shrinkage measurement in silicon solar cell by electroreflectance method, in Spectroscopic Characterization Techniques for Semiconductor Technology 111, Proceedings SPIE Vol. 946, O. J. Glembocki, F. H. Pollak, and F. Ponce, eds., SPIE, Bellingham, Washington (1988) pp. 76–81.
B. K. Janousek and R. C. Carscallen, Approaches to enhancing the sensitivity of direct coupled photoacoustic spectroscopy as applied to GaAs, in Spectroscopic Characterization Techniques for Semiconductor Technology, Proceedings SPIE Vol. 452, F. H. Pollak and R. S. Bauer, eds., SPIE, Bellingham, Washington (1987) pp. 121–127.
C. E. Jones, M. E. Boyd, W. H. Konkel, S. Perkowitz, and R. Braunstein, Noncontact electrical characterization of epitaxial HgCdTe, *J. Vac. Sci. Technol*. A **4**, 2056–2060 (1986).
Y. R. Lee, A. K. Ramdas, F. A. Chambers, J. M. Mcesc, and L. R. Ram Mohan, Piezomodulated electronic spectra of semiconductor heterostructures: GaAs/AIGaAs quantum well structures, in Spectroscopic Characterization Techniques for Semiconductor Technology, Proceedings SPIE Vol. 452, F. H. Pollak and R. S. Bauer, eds., SPIE, Bellingham, Washington (1987) pp. 105–110.
T. W. Nee, T. L. Cole, A. K. Green, M. E. Hills, C. K. Lowe-Ma, and V. Rehn, Infrared-wavelength modulation spectra of In-GaAs grown by MBE and LPE, in Spectroscopic Characterization Techniques for Semiconductor Technology, Proceedings SPIE Vol. 452, F. H. Pollak and R. S. Bauer, eds., SPIE, Bellingham, Washington (1987) pp. 142–151.
G. Niquet, J. F. Dufour, G. Chabrier, M. Q’Jani, and P. Vernier, Characterization by electroreflectance of thin films and thin film interfaces in layered structures, in Modern Optical Characterization Techniques for Semiconductors and Semiconductor Devices, Proceedings SPIE Vol. 794, O. H. Glembocki, F. H. Pollak, and J. J. Soong, eds., SPIE, Bellingham, Washington (1987) pp. 111–115.
P. Parayanthal, H. Shen, F. H. Pollak, O. J. Glembocki B.V. Shanabrook, and W.T. Beard, Photoreflectance of GaAs/GaAlAs multiple quantum wells: topographical variations in barrier height and well width, Appl. Phys. Lett. **48**, 1261–1263 (1986).
U. K. Reddy, G. Ji, R. Houdre, H. Unlu, D. Huang, and H. Morkoc, Study of GaAs/AIGaAs and InGaAs/GaAs multiple quantum wells grown on non-polar substrates by photoreflectance, in Modern Optical Characterization Techniques for semi-conductors and Semiconductor Devices, Proceedings SPIE Vol. 794, O. H. Glembocki, F. H. Pollak, and J. J. Soong, eds., SPIE, Bellingham, Washington (1987) pp. 116–120.
H. Shen, S. H. Pan, F. H. Pollak, and R. N. Sacks, Photoreflcctance and thermoreflectance of a GaAs/Ga_0.82_Al_0.18_As multiple quantum well, in Spectroscopic Characterization Techniques for Semiconductor Technology III, Proceedings SPIE Vol. 946, O. J. Glembocki, F. H. Pollak, and F. Ponce, eds., SPIE, Bellingham, Washington (1988) pp. 36–42.
H. Shen, Z. Hang, F. H. Pollak, K. Capuder, and P. E. Norris, *In situ* monitoring of OMVPE of GaAs and Ga_1−a_Al_1_As (*x* = 0.17) by contactlcss photoreflectance, in Surface and Interface Analysis of Microelectronic Materials Processing and Growth, Proceedings SPIE Vol. 1186, L. J. Brillson and F. II. Pollak, eds., SPIE, Bellingham, Washington (1989) pp. 27–35.
X. Yin, F. H. Pollak, J. T. Fitch, C. H. Bjorkman, and G. Lucovsky, Photoreflectance study of strain at Si/SiO_2_ interfaces prepared by thermal oxidation of silicon, in Surface and Interface Analysis of Microelectronic Materials Processing and Growth. Proceedings SPIE Vol. 1186, L. J. Brillson and F. H. Pollak, eds., SPIE, Bellingham, Washington (19S9) pp. 122–130.

## 6. Photoluminescence

### 6.1 Introduction

Photoluminescence (PL) depends on the fact that electrons residing in the valence band of a semiconductor can be excited via optical absorption to the conduction band, to an impurity, or to a defect level in the energy gap. PL can be used to determine the energy gap of a semiconductor sample. This technique is especially useful for III–V and II–VI ternary alloys like Al*_x_*Ga_1−_*_x_*As and Zn*_x_*Cd_1_*_−__x_*Te, because the energy gap, which varies with the compositional parameter x, must be accurately known for most applications. When this process is inverted, *x* can be found from the gap value and the known relation between gap energy and composition. Photoluminescence also detects the presence of impurities and crystalline defects in semiconductors, which affect materials quality and device performance. Each impurity produces a characteristic feature or set of featuresin the spectrum. Hence, the impurity type can be identified, and multiple impurities can be detected in a single spectrum. In some cases, PL can measure the concentration of impurities. Comparison of PL peak halfwidths from sample to sample gives an indication of impurity concentration, carrier concentration, and crystal perfection.

### 6 2 Physical Basis

Photoluminescence results from radiative relaxation of an optically excited population. In order to cause this excitation, the incoming photon energy must equal or exceed the energy difference between the initial and final states of the electron. Such an excited electron usually returns to its initial state after a short time. If the excited electron returns to its initial state by radiative means, the process emits a photon whose energy is the difference between the excited and the initial state energies. The spectral distribution of the emitted photons shows an emission peak at the energy (or wavelength) corresponding to each excited level.

Photoluminescence is complicated by the behavior of the electron during its excited period. The excited electron leaves behind it a deficiency in the valence band, a mobile hole. The Coulomb attraction between the excited electron and the hole can bind the two particles into a system called a free exciton, much as a proton and an electron form a bound hydrogen atom. The exciton can move as a unit through the crystal, but carries no current because its net charge is zero. From this perspective, the return of the electron to its initial state can be viewed as the collapse of the temporary excitonic state, when the electron recombines with the hole.

The exciton influences the PL spectrum in several ways. Because it is a bound state, the excited state energy is slightly less than the bandgap energy, generally by a few me V. Hence, for PL near the energy gap, the equation for the energy of the emitted photon is

$$\hbar \omega =E_{g}-E_{\mathrm{ex}},$$ (17)

where *E*_ex_ is the binding energy of the excitonic state. This equation applies for a direct energy gap semiconductor. For an indirect gap semiconductor, a phonon must also be involved to properly conserve momentum. Then the equation for the emitted photon energy is (E_pb_is the photon energy)

$$\hbar \omega =E_{g}-E_{\mathrm{ex}}-E_{\mathrm{ph}}.$$ (18)

However, this free exciton recombination dominates only when the sample is very pure. When donor, acceptor, or neutral impurities are present, free excitons respond to the Coloumb fields of these defects to form bound excitons. Each type or exciton produces a PL peak when recombination occurs, and each can be identified in the spectrum.

### 6.3 Experimental and Technical Details

**Fig. 16:**
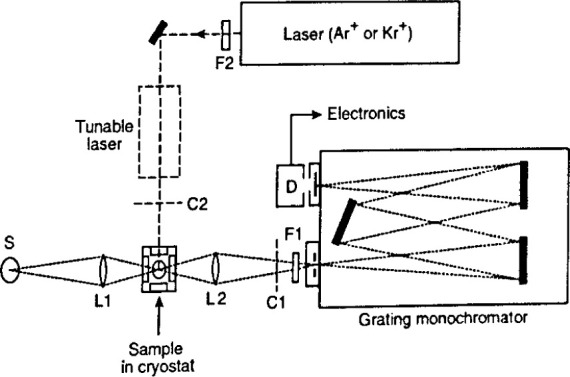
Schematic diagram of a photolumineseenee arrangement, showing the exciting Ar^*^ or Kr^*^ laser, filter F2 to block unwanted laser lines, the sample mounted in a cryostat, lens L2 to bring the PL radiation to the monochromator entrance slit, hopping wheel CI to modulate the light for lock-in detection, filter Fl to exclude the laser line from the monochromator, the grating monochromator itself, and detector D followed by appropriate electronics to process and analyze the signal The tunable dye laser and chopping wheel C2 shown in dashed outline are auxiliary equipment for lumineseence excitation measurements, a related technique. Lamp S and lens LI allow auxiliary absorption spectroscopy, using the same monochromator and detector. (See Sec. 6.5, General Refs., Lightowlers (1990), fig. 4, p. 138.)

Figure 16 shows the main elements of a standard PL arrangement. Any of several commercially available types of laser may be used, provided thatenergy of the laser’s photons exceeds the energy gap of the material, and the laser power is adequate to excite a usable PL signal. An argon ion laser is suitable for many semiconductors of technological interest such as Si (1.12 eV), Al*_x_*Ga_1−_*_x_*As (1.42 eV to 2.16 eV) and Zn*_x_*Cd_1−_*_x_*Te. Laser powers ≤50 mW are usually adequate, but power densities must be minimized to avoid sample heating effects. It is generally possible to avoid heating and still obtain adequate signal to noise by defocusing the laser or reducing the incident laser power.

The sample’s PL radiation passes through a monochromator and then to a detector, to yield the spectral distribution of intensity versus wavelength or energy. A standard photomultiplier tube is usually employed for visible and near visible applications. Quantum detectors such as germanium and InAs photodiodes are employed for the near infrared. All of these give the best signal-to-noise ratio when cooled to temperatures as low as 77 k for solid-state photodiodes. Improvement in sensitivity comes with the use of an optical multi-channel analyzer such a Si photodiode array. The array has the advantage of providing complete spectra in a short time.

In spectral regions where signal to noise is detector limited, decided improvements in sensitivity anddecreases in measurement times come if the grating monochromator in Fig. 16 is replaced by an Michelson interferometer to carry out Fourier transform spectroscopy. This instrument has already been discussed in the section on infrared spectroscopy.

It is usually necessary to cool the sample below room temperature to observe the best PL spectra. Cooling reduces the thermal broadening of the excited carrier spectrum of the order *k*_B_*T*, and also reduces the importance of nonradiative de-excitation processes. Cooling to liquid nitrogen temperature is often adequate. The sample can be mounted to a cold finger connected to a liquid nitrogen dewar, and can be held to within a few degrees of 77 K.

When necessary, cooling to liquid helium temperatures can be conveniently obtained by a continuous-flow liquid helium system. With proper shielding and an adequate flow rate of helium (typically 1 L/h to 3 L/h), sample temperatures as low as 6 K to 10 K can be maintained. Temperatures down to ∼10 K can be reached by mechanical refrigerators. If necessary, temperatures to 4 K can be obtained by immersion in liquid helium or to 2 K by pumped helium methods.

### 6.4 Illustrative Applications

**Fig. 17:**
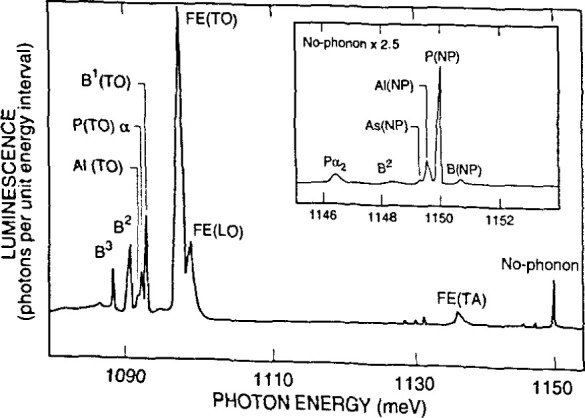
PL spectrum of high-resistivity (>20 kΩ cm) near-intrinsic silicon showing the presence of the following impurities and their concentrations, in units of 10^12^ cm^−3^: B, 1.36; P, 1.69; Al, 0.61; and As, 0.14. The fingerprint features for each element are marked. Free-exciton lines are marked FE. Because silicon is an indirect-gap semiconductor, phonon modes must be involved in FE transitions. They are indicated as TO (transverse optical), LO (longitudinaloptical), and TA (transverse acoustic). Peaks labeled NP (no phonon) come from bound excitons, which do not require phonon assistance. The technique for deriving quantitative impurity concentration data from such spectra is discussed in the caption for Fig. 18. (See Sec. 6.5, General Refs., Lightowlers (1990), fig. 9, p. 144.)

**Fig. 18:**
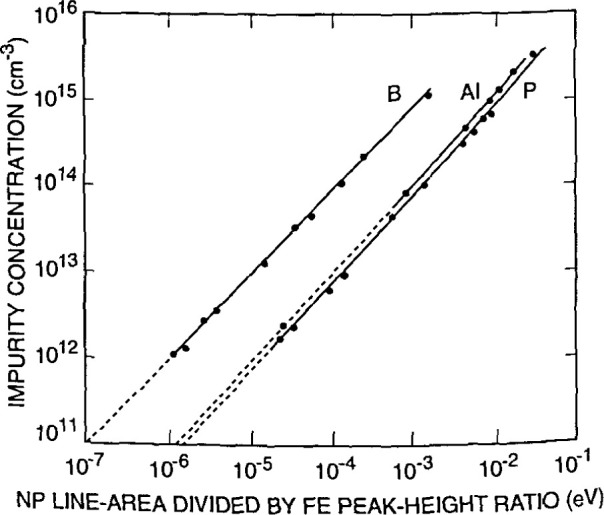
Calibration chart to convert PL information like that in Fig. 17 into impurity concentration for B, Al, and P in silicon. It is difficult to establish absolute intensity standards for PL, because of differences in laser excitation power and focusing, temperature, and other factors. This chart uses a calibration method which is internal to a given spectrum, and hence avoids many of the problems of absolute calibration, although it was established using careful independent measurements of concentrations, and of temperature and light intensity. The area of the NP peak for the particular impurity is ratioed against the height of the FE(TO) peak in the same spectrum. More recent work has extended the upper limit of the calibration curves to about 10^17^ cm^−3^. (See Sec. 6.5, General Refs., Lightowlers (1990), fig. 14, p. 148.)

**Fig. 19:**
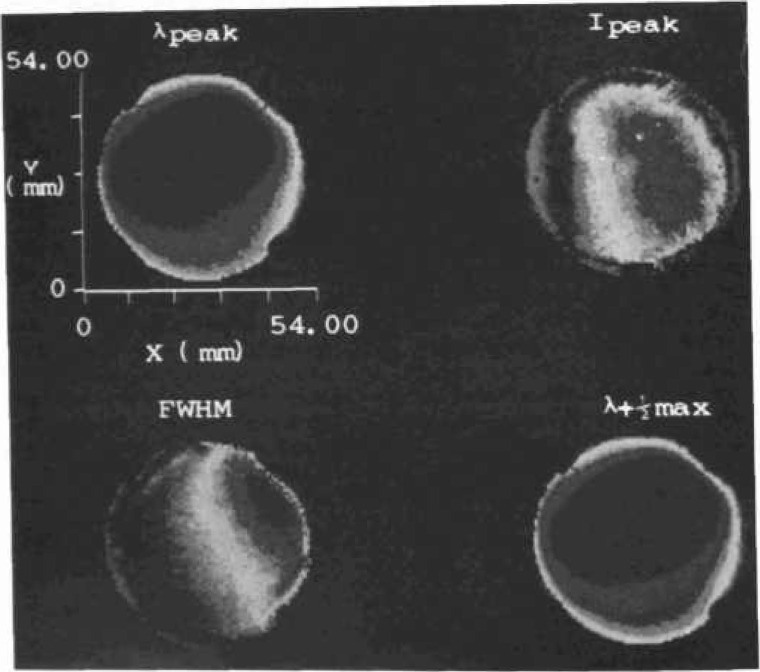
Two-dimensional maps of PL parameters from a 50 mm wafer of cpitaxial InGaAsP grown on InP, obtained using a commercial system with *x-y* scanning capability. Upper left, wavelength of the PL peak, which is related to sample composition; upper right, peak intensity, related to defect density; lower left, peak full width at half maximum (FWHM), related to closeness of the lattice match between layer and substrate; lower right, upper wavelength at which PL intensity falls to 50 % of the peak value, related to sample composition. Spatial variations inall the parameters arc clearly seen. (Sec Sec. 6.5, Applications Refs., Hcnncssy, Miner, and Moore (1990), fig. 3.)

Figure 17 shows how specific impurities in a semiconductor such as silicon clearly appear in PL spectra. The caption explains the source of each peak. Figure 18 illustrates the conversion of PL data into accurate values for impurity concentrations. Figure 19 shows how two-dimensional PL mapping can help evaluate the homogeneity and quality of a semiconductor wafer, in this case an epitaxial layer of InGaAsP grown on InP. The technique uses the fact that each parameter that describes a PL peak can be related to a sample property. The peak position, for instance, gives the energy gap value, which for an alloy like InGaAsP varies with the proportions of the component elements. Hence, a map of peak PL wavelength correlates well with a map of composition.

Table 6 presents the sensitivities of typical quantities measured by photoluminescence such as composition in III–V and II–VI alloys and the concentration of B, P, As, and Al in Si. The reader should refer to the citations in the table for more specific details.

### 6.5 References

#### General

P. Goldberg, Luminescence of Inorganic Solids, Academic, New York (1966).
E. C Lightowlcrs, Photoluminesccnce characterisation, in Growth and Characterization of Semiconductors, R. A. Stradling and P. C Klipstein, cds., Adam Hilger, Bristol (1990) pp. 135–163.
D. K. Schroder, Semiconductor Material and Device Characterization, John Wiley, New York (1990) pp. 490–494.
M. L. W. Thewalt, M. K. Nissen, D. J. S. Beckett, and K. R. Lundgren, High performance photoluminescence spectroscopy using Fourier transform interferometry, in Impurities, Defects and Diffusion in Semiconductors: Bulk and Layered Structures, Materials Research Society Symposia Proceedings Vol. 163, D. J. Wolford, J. Bernholc, and E. E Haller, cds., Materials Research Society, Pittsburgh, Pennsylvania (1989) pp. 221–232.

#### Applications

K. K. Bajaj and D. C. Reynolds, An overview of optical characterization of semiconductor structures and alloys, in Modern Optical Characterization Techniques for Semiconductors and Semiconductor Devices, SPI E Proceedings Vol. 794, O. J- Glem-bocki, F. H. Pollak, and J. J. Song, cds., SPIE, Bellingham, Washington (1987) pp. 2–10.
S. G. Bishop, Characterization of semiconductors by photolumincsccnce and photolumincsccnce excitation spectroscopy, in Optical Characterization Techniques for Semiconductor Technology, SPIE Proceedings Vol. 276, D. E. Aspnes, S. So, and R. F. Potter, cds., SPIE, Bellingham, Washington (1981) PP-2–10.
M. Bugajaski, K. Nauka, S. S. Rosner, and D. Mars, Photolumincsccnce studies of annealed GaAs films grown on Si su strates, in Heterocpitaxy on Silicon: Fundamentals, Structure, and Devices, Materials Research Society Symposia Proceedings Vol. 116, H. K. Choi, R. Hull, H. Ishiwara, and R. J. Nemanich, cds., Materials Research Society, Pittsburgh, Pennsylvania (1988) pp. 233–238.
Y. H. Chen and S. A. Lyon, Photoluminescence and diffusivity of free excitons in doped silicon, IEEE J. Quantum Electron. QE-25, 1053–1055 (1989).
H. Conzelmann, Photoluminescence of transition metal complexes in silicon, Appl. Phys. A 42, 1–18 (1987).
W. M. Duncan, M. L. Eastwood, and H-L. Tsai, Fourier transform photoluminescence analysis of trace impurities and defects in silicon, in Materials Characterization, Materials Research Society Symposia Proceedings Vol. 69, N. Cheung and M.-A. Nico-let, eds., Materials Research Society, Pittsburgh, Pennsylvania (1986) pp. 225–230.
J. A. Fouquet, R. R. Saxena, and G. A. Patterson, Near-infrared photoluminescence of high-resistivity epitaxial GaAs and InP and of epitaxial GaAs on Si, IEEE J. Quantum Electron. QE-25, 1025–1034 (1989).
A. Freundlich, G. Neu, A. Leycuras, R. Carles, and C. Verie, Heterogeneous strain relaxation in GaAs on Si (100), in Heteroepitaxy on Silicon: Fundamentals, Structure, and Devices, Materials Research Society Symposia Proceedings Vol. 116, H. K. Choi, R. Hull, H. Ishiwara, and R. J. Nemanich, eds., Materials Research Society, Pittsburgh, Pennsylvania (1988) pp. 251–256.
J. Hennessy, C. Miner, and C. Moore, Photoluminescence mapping in inspection and process control, Photonics Spectra **24**, 91–96 (1990).
E. D. Jones and L. R. Dawson, Photoluminescence studies of ln-GaAlAs quaternary alloys, in Spectroscopic Characterization Techniques for Semiconductor Technology III, Proceedings SPIE Vol. 946, O. J. Glembocki, F. H. Pollak, and F. Ponce, cds., SPIE, Bellingham, Washington (1988) pp. 172–176.
E. S. Koteles, Y. Y. Chi, and R. F. Holmstrom, Low temperature photoluminescence signature of a two-dimensional electron gas, in Modern Optical Characterization Techniques for Semiconductors and Semiconductor Devices, Proceedings SPIE Vol. 794, O. J. Glembocki, F. H. Pollak, and J. J. Song, eds., SPIE, Bellingham, Washington (1987) pp. 61–65.
H. P. Lee, Y-H. Huang, X. Liu, J. S. Smith, E. R. Weber, P. Yu S. Wang, and Z. Lilicnthal-Weber, The photoluminescence and TEM studies of patterned GaAs films on Si substrates grown by molecular beam epitaxy, in Heteroepitaxy on Silicon: Fundamentals, Structure, and Devices, Materials Research Society Symposia Proceedings Vol. 116, H. K. Choi, R. Hull, H. Ishiwara, and R. J. Ncmanich, cds., Materials Research Society, Pittsburgh, Pennsylvania (1988) pp. 219–226.
C. J. Miner, Non-destructive, whole wafer assessment of optoelectronic epitaxial materials, Semicond. Sci. Techno.7, A10–A15 (1992).
C. J. L. Moore and C. J. Miner, A spatially resolved spectrally resolved photoluminescence mapping system, J. Crystal Growth **103**, 21–27 (1990).
A. L. Moretti, F. A. Chambers, G. P. Devane, and F. A Kish, Characterization of GaAs/Al_x_Ga_1−x_As structures using scanning photolumincsccnce, IEEE J. Quantum Electron. **QE-25**, 1018–1024 (1989).
R. J. Ncmanich, D. K. Bicgclscn, R. A. Street, B. Downs, B. S. Krusor, and R. D. Yingling, Strain in graded thickness GaAs/Si hctcrocpitaxial structures grown with a buffer layer, in Heteroepitaxy on Silicon: Fundamentals, Structure, and Devices, Materials Research Society Symposia Proceedings Vol. 116, II. K. Choi, R. Hull, H. Ishiwara, and R. J. Ncmanich, eds., Materials Research Society, Pittsburgh, Pennsylvania (1988) pp. 245–250.
T. Nishino, H. Nakayama, J. Katsura, and Y. Hamakawa, Photolumincsccnce characterization of thermally induced defects in Czochralski-grown Si wafers, Optical Characterization Techniques for Semiconductor Technology, Proceedings SPIE Vol. 276, D. E. Aspncs, S. So, and R. F. Potter, eds., SPIE, Bellingham, Washington (1981) pp. 31–38.
T. Nishino, A new high-sensitivity characterization method of interface stress at hctcrostructurcs by Cr-related luminescence, IEEE J. Quantum Electron. **QE-25**, 1046–1052 (1989).
J. E. Potts, T. L. Smith, and H. Cheng, Photolumincsccnce studies of donors in molecular beam epitaxy (MBE) grown ZnSc, in Modern Optical Characterization Techniques for Semiconductors and Semiconductor Devices, Proceedings SPIE Vol. 794, O. J. Glembocki, F. H. Pollak, and J. J. Song, eds., SPIE, Bellingham, Washington (1987) pp. 27–33.
D. C. Reynolds and C. W. Litton, Semiconductor materials characterization by high-resolution optical spectroscopy, in Optical Characterization Techniques for Semiconductor Technology, Proceedings SPIE Vol. 276, D. E. Aspncs, S. So, and R. F Potter, cds., SPIE, Bellingham, Washington (1981) pp. 11–30.
E. K. Ricmcr T. G. Stoebe, and A. A. Khan, Scanning photolumincsccnce, in Modern Optical Characterization Technique, for Semiconductors and Semiconductor Devices, Proceedings SI E Vol 794 O. J. Glembocki, F. II. Pollak, and J. J. Song, eds., SPIE, Bellingham, Washington (1987) pp. 20–26.
B. J. Skromme, R. Bhat, H. M. Cox, and E. Colas, Identification of donors in GaAs by resonantly excited high fild magnctospcc troscopy, IEEE J. Quantum Electron. **QE-25**, 1035–1045 (1989).
M. L. W. Thcwalt and D. M. Brake, Ultra-high resolution photo-lumincsccncc syudies of bound excitions and multi bound exction complexes in silicon, Materials Science Forum Vols. 65.66 (1990) 187–198.
B. A. Wilson, Novel applications of optical tcchniques to the study of buricd semiconductor interfaccs, IEEE J. Ouantum Elcctron. **QE-25**, 1012–1017 (1989)
S. Zemon, C. Jaganna,. S.K. Shastry, W.J. Miniscalco and G. Lambcrt, Resonant photoluminescencc excitation of anncaled GaAs films grown on Si substrates, in Heterocpitaxy on Silicon Fundamcntals, Structure, and Devices Materials Research Society Symposia Procedings Vol. 116. 11. K. Choi. R. IIull. II. Ishiwara, and R.J. Ncmanich, cds., Matcrials Research Socicty, Pittsburgh, Pennsylvania (1988) pp. 239–245.

## 7. Raman Scattering

### 7.1 Introduction

Raman scattering results when photons interact with optica] lattice vibrations (phonons) of a semiconductor crystal lattice. The way in which these phonons appear in a Raman spectrum depends on the crystallinity of a sample and on its crystal orientation. Hence, Raman scattering can determine whether a sample is amorphous or crystalline, and whether the crystal is of good quality or is altered by damage or imperfections. The method is also sensitive to strain effects, which change semiconductor lattice structure and hence phonon frequencies. Since phonon frequencies and amplitudes in an alloy semiconductor like Al*_x_*Ga_1−_*_x_*As change with the degree of alloying, Raman scattering can be used to measure composition as well. By changing the wavelength of the light exciting the scattering, the penetration depth can be changed, which gives the capability to probe layered or inhomogeneous structures.

In microprobe Raman scattering, a microscope is coupled to the Raman system, making it possible to probe regions as small as ~1*µ*m across. This allows for the identification of contaminating impurities in extremely small regions of the specimen. In resonance Raman scattering, the scattering process is strengthened when the incoming photon energy matches the energy gap or other higher-order critical point energies in the sample’s band structure. This resonance strengthens the inherently weak Raman process and also gives band structure information as well as phonon information.

### 7.2 Physical Basis

Raman scattering, a two-photon process, is more complex than one-photon optical processes such as photoluminescence. If light impinges on the surface of a semiconductor, a large portion is reflected, transmitted, absorbed, or elastically scattered (Ray-leigh scattering), with no change in frequency A small part of the light interacts inelastically with phonon modes, so that the outgoing photons have frequencies shifted from the incoming values These are the Raman-scattered photons. Since the photons can either gain energy or lose energy in their phonon interactions, the scattered light can be of higher frequency (anti-Stokes-shifted) or of lower frequency (Stokes-shifted) than the incident light. Because of statistical considerations the Stokes modes are stronger and are usually the ones observed in Raman measurements at room temoerature.

The up-shifted and down-shifted photons can be treated as side bands of the exciting light that come from the nonlinear interaction between the light and the material. This can be seen by examining the crystal polarization P due to the phonons, which is given by

P=αE (19)

where*E* is the applied electric field E_0_ coswf and cosωt and the polarizability *α*is given by

$$\alpha =\alpha_{1}u+\alpha_{2}u^{2}+\alpha_{3}u^{3}+\ldots ,$$ (20)

where *u* is the phonon displacement, and *α*_1_, *α*_2_,… are constants. The first term in Eq. (20) is the dipole approximation, and the other terms represent more complex anharmonic contributions. If the phonon vibrates at frequency *Ω, u* is of the form *u* = *u*_0_ cos*(Ωt)* and Eq. (19) becomes

$$\begin{array}{l} P=E_{0}[\alpha_{1}u_{0}\;\cos\;\left(\omega t\right)\;\cos\;\left(\Omega t\right)+\\ \alpha_{2}u_{0}^{2}\;\cos\;\left(\omega t\right)\;{\;\cos}^{2}\;\left(\Omega t\right)+\ldots ]. \end{array}$$ (21)

From standard trigonometric identities, Eq. (21) can be rewritten as expressions that contain cos(*ω+Ω)t*, cos*(ω±*2*Ω)t* …, cos*(ω±nΩ)t.* The leading term cos(*ω±Ω)t* represents the fundamental Raman-shifted bands at frequency *ω+Ω*(so-called anti-Stokes lines) and *ω−Ω*(Stokes lines), and the others represent the interaction of the photon with multiple phonons (*n* = 2, 3, …).

This simple development of the theory gives the shift in photon frequency, that is, where the Raman bands lie relative to the exciting wavelength. However, the intensity and line shape of the Raman bands are more difficult to calculate. Although some appropriate theory exists, it is not easy to apply to specific semiconductors. In general, Raman scattering is a weak process, and the higher order terms in Eq. (20) generally contribute weakly. Beyond these qualitative trends, line-shape analysis of Raman spectra of semiconductors is not well developed. However, it is true that smaller half-widths correlate with higher levels of sample crystalline perfection.

When carried out in detail, the calculation of the Raman intensity depends strongly on the orientation and polarization of the exciting light relative to the crystal axes, since such geometric considerations determine how the field and the polarization interact. Hence, the Raman spectrum from a given crystal depends on its orientation, with various “allowed” and “forbidden” Raman modes for different orientations.

An exception to the statement that Raman scattering is weak occurs in resonant Raman scattering. If the energy of the exciting photon is chosen to match a fundamental feature in the semiconductor band structure, such as the energy gap or higher order critical points in the band structure, the amount of energy imparted to the lattice increases dramatically, and so does the strength of the Raman modes.

### 7.3 Experimental and Technical Details

The overriding consideration in Raman scattering is the weakness of the signal, and the difficulty of separating it from unavoidable accompanying spectral information. Weak Raman peaks usually must be measured in the immediate neighborhood of intense Rayleigh scattering, which occurs at the energy of the exciting photon. Raman measurements require the strongest possible light sources that will not damage the sample, special optical methods to filter out the Rayleigh peak, and sensitive detection schemes to capture the few emerging Raman-shifted photons.

Any monochromatic light source can act as a Raman source. Most often, lasers operating at visible frequencies are employed to provide the necessary power. To give flexibility in varying the penetration depth, and some capability to excite resonance Raman scattering, it is better to use lasers that can be tuned over several powerful lines in the visible. Argon-ion and krypton-ion lasers are good choices; they are powerful, commercially available, and easily tunable, one from the ultraviolet to the green, the other toward the red end of the spectrum. For maximum flexibility in tuning, say for exact resonant coincidence, a tunable dye laser is the best choice. With the range of available dyes, these lasers can be tuned through the energy gaps of most semiconductors. The power is much lower than that of ion lasers but the increased signal due to the resonant effect more than compensates tor this.

The optical arrangement for Raman spectroscopy is similar to that for photoluminescence (fig. 16), but with one important exception: a single monochromator is usually inadequate to separa the Raman signal from the strong accompanying Rayleigh light. A double monochromator is standard, consisting of two tandem gratings turning to gether and sequentially dispersing the light. In recent years, holographic notch filters have matured sufficiently that they can be used to elminate the Rayleigh signal and allow the use of a single monochromator. Triple monochromators are sometimes used.

Raman scattering excited in the visible or ultraviolet can be detected by visible or ultraviolet detectors, so photomultiplier tubes (PMT) and array detectors work well. The PMTs should be chosen to give broad spectral coverage and to display a low dark count for maximum sensitivity. Cooled operation to reduce dark count is also important. As in photoluminescence work, it is possible to use Fourier methods to enhance Raman sensitivity or to reduce data collection time.

### 7.4 Illustrative Applications

**Fig. 20:**
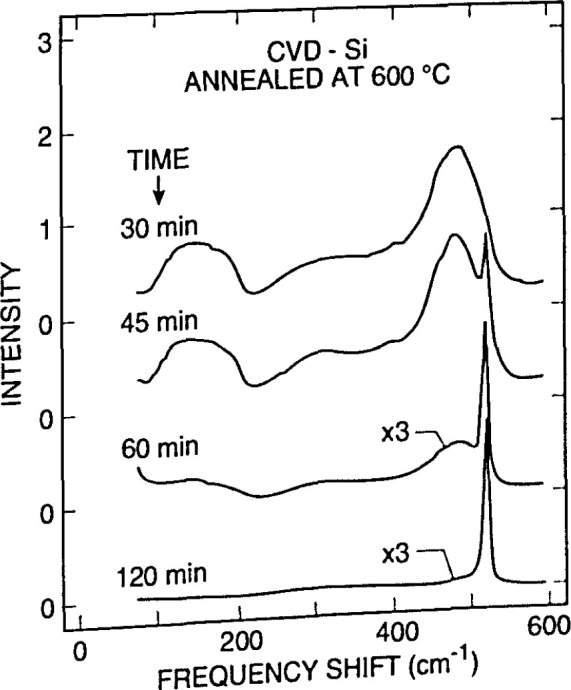
Raman speetra of silicon grown by chemical sapor deposition showing change in the charaeter of the silicon after annealing. Teh top speetrum, taken after 30 min of anncaling is virtually the same as from as grown matcrial. It shows only broad peaks coming from amorphous silieon. After anncaling for 45 min, a sharp line appears at 520 cm^−1^, which comes from the optical phonon charaeteristie of erystalline silicon. The broad structure continues to decrease with annealing time, until after 120 min, the speetrum indieates that little or no amorphous silicon remains. (see see. 7.5, General Refs, Nemanich (1986). fig. 1. p 26

**Fig. 21:**
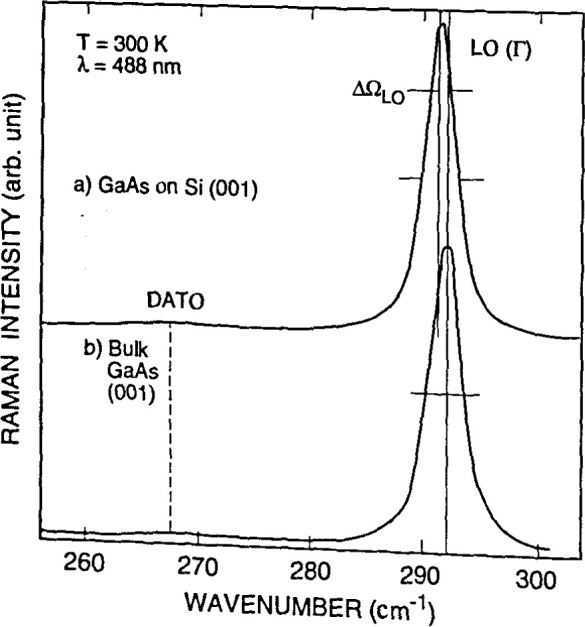
Raman spectra of bulk (100) GaAs (lower curve) compared to that from a GaAs film 2 *µ*m thick grown on (100) silicon (upper curve). The main peak near 292 cm^−1^ comes from the longitudinal optical (LO) phono, Peak halfwidths are the same in both curves. indicating similar sample quality. The barely visible disorder-activated transverse optical (DATO) peak remains equally small in both spectra, also indicating that there is no signifieant disorder. However, the peak position for the film is 0.7 cm^−1^ lower than for the bulk sample, because the epilayer is stressed. Raman methods can easily detect and measure such small changes in peak position. (See See. 7.5, Applications Refs., Freundlich, Neu, Leycuras, Carles, and Verie (1988), fig. 1, p. 252.)

**Fig. 22:**
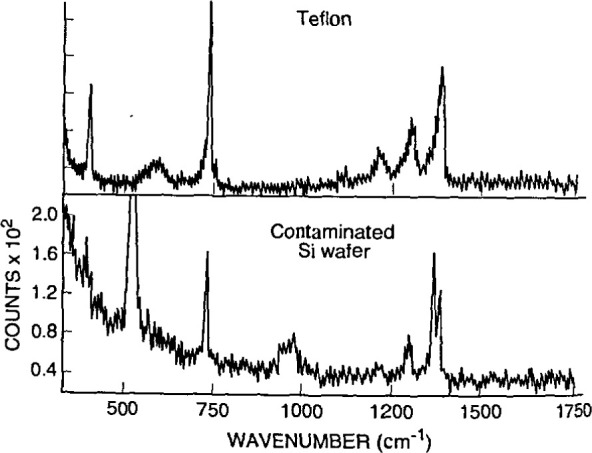
Raman microprobe spectrum of a silicon wafer with surface contamination (lower plot) compared to the Raman spectrum of polytctrafluorethylene or teflon (upper plot). The peaks in the lower spectrum at 520 cm^−1^ and 950 cm^−1^ are known phonon modes for silicon. The additional peaks arise from the contaminant. They resemble the teflon spectrum and suggest that the contamination came during wafer processing, which included polishing in a slurry containing organic solvents, and etching in a CF_4_/H_2_ plasma. Polymer could have been deposited on the wafer either as teflon from a container holding the slurry, or during the plasma etch. (Sec Sec. 7.5, Applications Refs., Adar (1986), fig. 2, p. 234.)

The sensitivity of Raman scattering to phonon modes makes it possible to distinguish between amorphous and crystalline semiconductors. Figure 20 illustrates the direct way in which Raman spectra follow the increasing presence of crystalline silicon, as annealing proceeds on amorphous material. Figure 21 shows how strain in a semiconductor appears in a Raman spectrum. The Raman peak for epitaxial GaAs grown on a silicon substrate shifts relative to that for bulk GaAs, because the epilayer lattice spacing changes under the strain induced by the mismatch between film and substrate. Micro-probe Raman analysis can be helpful for examining contamination by small particles or for examining thin films, as illustrated in Fig. 22.

Table 7 gives the sensitivities of typical quantities measured by Raman spectroscopy such as stress, impurity concentrations of C and Zn in GaAs, alloy composition, and temperature. For more specific details, the reader should refer to the citations in the table.

#### General

Light Scattering in Solids I. Introductory Concepts, M. Cardona, ed., Springer-Verlag, Berlin (1983).
Light Scattering in Solids II. Basic Concepts and Instrumentation, M. Cardona and G. Guntherodt, eds., Springer-Verlag, Berlin (1982).
G. J. Exarhos, Molecular characterization of dielectric films by laser Raman spectroscopy, in Characterization of Semiconductor Materials, G. E. McGuire, ed., Noyes, Park Ridge, New Jersey (1989) pp. 242–288.
J. Geurts and W. Richter, Raman scattering from interface regions; structure, composition, and electronic properties, in Semiconductor Interfaces: Formation and Properties, G. Le Lay, J-Derrien, and N. Boccara, eds., Springer-Verlag, Berlin (1987) pp. 328–334.
R. J. Nemanich, Raman spectroscopy for semiconductor thin film analysis, in Materials Characterization, Materials Research Society Symposia Proceedings Vol. 69, N. Cheung and M-A-Nicolct, eds., Materials Research Society, Pittsburgh, Pennsylvania (1986) pp. 23–37.
J. Sapriel, Raman characterization of semiconductor superlat-tices, in Spectroscopic Characterization Techniques for Semiconductor Technology III, Proceedings SPIE Vol. 946, O. J. Glembocki, F. H. Pollak, and F. Ponce, eds., SPIE, Bellingham, Washington (1988) pp. 136–145.
B. Schrader, Possibilities and limitations of FT-Raman spectroscopy, in Practical Fourier Transform Infrared Spectroscopy, J. R. Ferraro and K. Krishnan, eds., Academic Press, San Diego, California (1990) pp. 167–202.

#### Applications

F. Adar, Application of the Raman microprobc to analytical problems of microelectronics, in Microelectronic Processing: Inorganic Materials Characterization, ACS Symposia Scries 295, L. A. Casper, ed., American Chemical Society, Washington, DC (1986) pp. 230–239.
F. Adar, Developments of the Raman microprobe instrumentation and applications, Microchemical Journal **538**, pp. 50–79 (1988).
P. M. Amirtharaj, K-K. Tiong, and F. H. Pollak, Raman scattering in Hg_0.8_Cd_0.2_Te, J. Vac. Sci. Technol. Al, 1744 (1983).
S. J. Chang, M. A. Kallel, K. L Wang, and R. C. Bowman, Strain distribution of MBE grown GcSi/Si layers by Raman scattering, in Spectroscopic Characterization Techniques for Semiconductor Technology III, Proceedings SPIE Vol. 946, O. J. Glembocki, F. H. Pollak, and F. Ponce, eds., SPIE, Bellingham, Washington (1988) pp. 163–168.
Z. C Feng, S. Perkowitz, and O. Wu, Raman and resonant Raman scattering from the HgTc/CdTe superlattice Phvs Rev B **41**,6057–6060 (1990).
Z. C Feng, S. Perkowitz, T. S. Rao, and J. B. Webb, Raman charactenzation of InSb/GaAs grown by metalorganic magnetron sputtering, in Layered Structures: Hcteroepitaxy, Supcr-lattices, Strain, and Mctastability, Materials Research Society Symposia Proceedings Vol. 160, B. D. Dodson, L J. Schowalter, J.E. Cunningham, and F. H. Pollak, eds., Materials Research Society, Pittsburgh, Pennsylvania (1990) pp. 739–744.
Z. C. Feng, S. Perkowitz, T. S. Rao, and J. B. Webb, Resonance Raman scattenng from epitaxial InSb films grown by mctalor-ganic magnetron sputtering, J. Appl. Phys. **68**, 5363–5365 (1990).
A. Freundlieh, G. Neu, A. Leycuras, R. Carles, and C Verie Heterogeneous strain relaxation in GaAs on Si (100) in Hcteroepitaxy on Silicon: Fundamentals, Structure, and Devices, Materials Research Society Symposia Proceedings Vol. 116, H. K. Choi, R. Hull, H. Ishiwara, and R.J. Nemanich, eds., Materials Research Society, Pittsburgh, Pennsylvania (1988) pp. 251–256
J. Gonzalez-Hernandez, D. Martin and R Tsu, Raman study of strain and microadhesion in silicon, in Spectroscopic Characterization Techniques for semiconductor Technology, Proceedings SpIE Vol. 452, F.H. Pollal and R.S. Bauer, eds., SPIE, Bellingham, Washington (1983) pp. 44–50.
D. Kirillov, P. Ho and G. A. Davis, Raman scattering study of mixing of GaAs/AlAS superlattices by ion implantation and rapid thermal anncaling, in Materials Characterization, Materials Research Society Symposia Proceedings Vol. 69, N. Cheung and M.-A. Nicolet, eds., Materials Research Society, Pittsburgh, pennsylvania (1986) pp. 185–190.
D. S. Knight and W. B. White, Characterization of diamond films by Raman spectroscopy, J. Mater. Res. **4**, 385–393 (1989).
P. Lao, W. C. Tang, A. Madhukar, and P. Chen, Alloy disorder effects in molecular beam epitaxially grown AIGaAs examined via Raman and Raylcigh scattering and near edge luminescence, in Spectroscopic Characterization Techniques for Semiconductor Technology III, Proceedings SPIE Vol. 946, O. J. Glembocki, F H. Pollak, and F. Ponce, eds., SPIE, Bellingham, Washington (1988) pp. 150–154.
J. Menendez, A. Pinczuk, J. P. Valladares, L. N. Pfeiffer, K. W. West, A. C. Gossard, and J. H. English, Resonance Raman scattering in short period GaAs-AIAs supcrlattices, in Spectroscopy of Semiconductor Microstructures, G. Fasol, A. Fasolino, and P. Lugli, eds., Plenum Press, New York (1989) pp. 157–164.
S. Nakashima and M. Hangyo, Characterization of semiconductor materials by Raman microprobe, IEEE J. Quantum Electron. QE-2S, 965–975 (1989).
R. J. Nemanich, D. K. Biegelscn, R. A. Street, B. Downs, B. S. Krusor, and R. D. Yingling, Strain in graded thickness GaAs/Si heterocpitaxial structures grown with a buffer layer, in Het-eroepitaxy on Silicon: Fundamentals, Structure, and Devices, Materials Research Society Symposia Proceedings Vol. 116, H. K. Choi, R. Hull, H. Ishiwara, and R. J. Nemanich, eds., Materials Research Society, Pittsburgh, Pennsylvania (1988) pp. 245–250.
R. J. Ncmanich, R. W. Fiordalice, and H. Jeon, Raman scattering characterization of titanium silicide formation, IEEE J. Quantum Electron. **QE-25**, 997–1002 (1989).
G. D. Pazionis, H. Tang, and I. P. Herman, Raman microprobe analysis of temperature profiles in CW laser heated silicon microstructures, IEEE J. Quantum Electron. **QE-25**, 976–987 (1989).
L. S. Plano and F. Adar, Raman spectroscopy of polycrystalline diamond films, in Raman and Luminescence Spectroscopy in Technology, Proceedings SPIE Vol. 822, J. E. Griffiths and F. Adar, eds., SPIE, Bellingham, Washington (1987) pp. 52–56.
C. J. Radens, B. Roughani, H. E. Jackson, J. T. Boyd, and R. D. Burnham, Raman microprobe analysis of strain induced by patterned dielectric films on GaAIAs structures, IEEE J. Quantum Electron. **QE-25**, 989–992 (1989).
B. Roughani, J. J. Jbara, J. T. Boyd, T. D. Mantei, and H. E. Jackson, Reactive ion etching of MBE GaAs; a Raman scattering study, in Spectroscopic Characterization Techniques for Semiconductor Technology III, Proceedings SPIE Vol. 946, O.J. Glembocki, F. H. Pollak, and F. Ponce, eds., SPIE, Bellingham, Washington (1988) pp. 146–149.
B. Roughani, H. E. Jackson, J. J. Jbara, T. D. Mantel, G. Hickman, C. E. Stutz, K. R. Evans, and R. L. Jones, Raman scattering studies of reactive ion-ctched MBE (100) n-type GaAs, IEEE J-Quantum Electron. **QE-25**, 1003–1007 (1989).
J. C. Tsang and S. S. Iycr, Raman spectroscopy and the characterization of buried semiconductor layers, IEEE J. Quantum Electron. **QE-25**, 1008–1011 (1989).
J. Wagner and M. Ramstener, Raman spectroscopic assessment of Si and Be local vibrational modes in GaAs layers grown by molecular beam epitaxy, IEEE J. Quantum Electron. **QE-25**, 993–996 (1989).

## Figures and Tables

**Fig. 14a:**
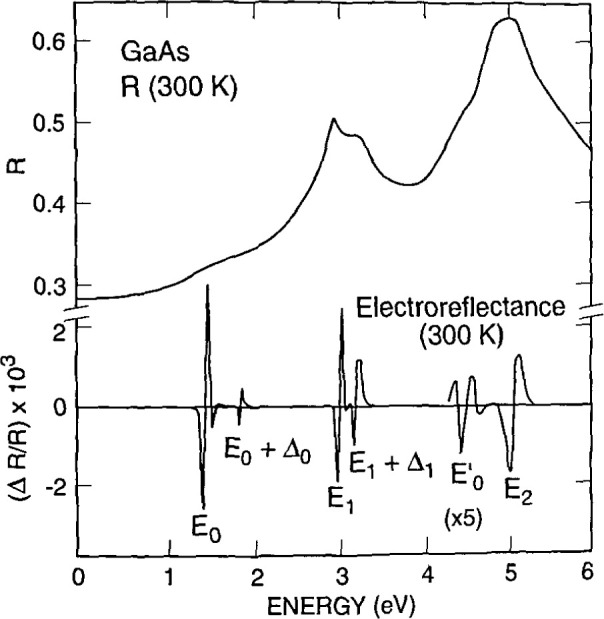
Conventional interband reflectivity spectrum for GaAs at 300 K, compared to the interband *∆R/R* spectrum at 300 K obtained by etcctroreflectance. The broad features in the plot for reflectivity *R*, such as the shoulder at the gap energy *E*_0_, become obvious sharp lines in the *∆R/R* data which lie on a baseline of zero signal. Structure at *E*_0_+*∆*_0_ which was invisible in the reflectivity spectrum is apparent in the *∆R/R* curve. (See Sec. 5.5, General Refs., Pollack and Glembocki (1988), fig. 1, p. 25.)

**Fig. 14b:**
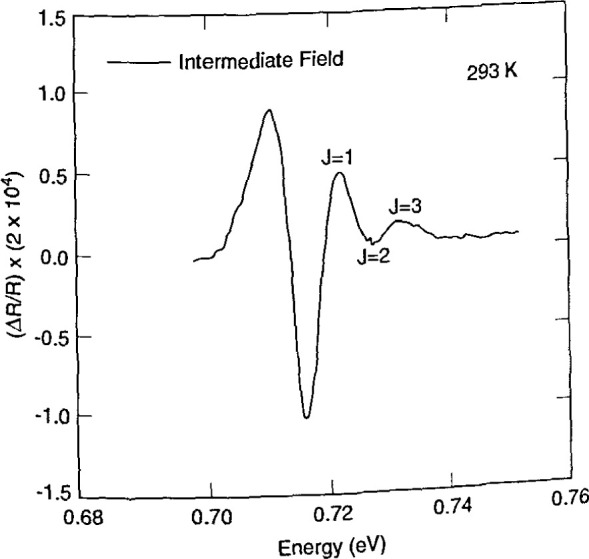
Photoreflcctance spectra for the intermediate field case for a moderately doped sample of InGaAs/InP with labeled case for a moderately doped sample of InGaAs/InP with labeled extrema (*J*= 1, 2, 3).

**Table 1:** Semiconductor quantities (horizontal rows) and optical characterization methods (vertical columns, labeled as follows: ELL, ellipsometry; IR, infrared spectroscopy; MIC, microscopy; MOD, modulation spectroscopy; PL, photolumineseenee; and RAM, Raman scattering). A bullet at the intersection of a given row and column means that the parameter can be determined by that technique using conventional methodology. Further details arc given in the discussion

	ELL	IR	MIC	MOD	PL	RAM
Carrier density	•^a^				•^a^	
Carrier mobility		•^a^				•^a^
Carrier scattering time	•			•^b^		
Composition	•	•	•	•	•	•
Crystal orientation						•
Crystallinity	•				•	•
Defects		•	•		•	•
Energy gap		•		•	•	
Film thickness	•	•	•			
Impurities		•	•	•	•	•
Resistivity		•				•
Stress	•	•		•	•	•

a If the effective mass is known.

b Time resolved.

**Table 2:** Conversion factors for units of measure

λ/nm	= 10^3^λ/μm
*E_λ_/*eV	= 1.2397/(λ/μm)
	= 1.2397 × 10^−4^λ^−1^/cm^−1^
λ^−1^/em^−1^	= 10^4^/(λ/μm)

**Table 3:** Spectral ellipsometry sensitivity. Given are sensitivities for the measurement of thicknesses, composition, damage profile, and temperature in a number of systems. The notations used for the measured quantity column are the complex reflection ratio, *ρ*; the amplitude ratio, *ψ*; and the phase shift, *⊿*

Method	Matrix	Quantity	Measured quantity	Conversion	Sensitivity^a^	Ref. (see below)
SE	SiO_2_/Si	SiO_2_ thickness	*ρ*	Fresnel Equations^[1]^ and Estimator^[2]^ (FE&E)	±0.2 Åto ±0.6 Å @1000 Åto 2300 Å	3
SE	SiO_2_/SiO_2_+Si/Si	Interfacial SiO_2_+Si thickness	*ρ*	FE&E and EMA^[4]^	±2Å@7Å	4
SE	SiO_2_/SiO_2_+Si/Si	SiO_2_ thickness	*ρ*	FE&E and EMA	(1112.1 ± 0.2) Å and (276.9 ± 0.2) Å	5
SE	SiO_2_/a-Si/c-Si+SiC/SiC/c-Si	Layer thicknesses	*ρ*	FE&E and EMA	±2 Åto ± 25 Å	6
SE	Polysilicon	Polysilicon composition (e.g., void+c-Si+a-Si)	*ρ*	FE&E and EMA	c-Si: 0.14 ± 0.02 void: 0.25 ± 0.06	7
SE	Si	Damage profile	*ρ*	FE&E and EMA	Damage range (170 ± 50) Åto (320±20)Å	8
SE	SiO_2_/polysilicon/SiO_2_/Si	Polysilicon and SiO_2_ thicknesses	*ρ*	FE&E and EMA	Native oxide (15 ± 0.3) Å; Poly ± 0.3 Åto ± 3.9 Å @ (240 to 1030) Å; Oxide ±0.4 Åto+1.7Å @(50 to 130) Å	9
SE	SiO_2_/Si/SiN/Si	Native oxide, Si, and nitride thickness	*ρ*	FE&E and EMA	**−**	10
SWE	SiO_2_/Si	Temperature	*ψ*, ⊿	polynomial	±10°C	11
VASE	Oxide/GaAs/Al_x_Ga_1−_*_x_*As/GaAs	Thicknesses and AIGaAs composition	*ρ*	FE&E and EMA	Oxide (34±3)Å GaAs(159±8)Å AIGaAs (865 ± 14) Å AIGaAs, *x* = 0.35 ± 0.02	12
VASE	Oxide/GaAs/Al*_x_*Ga_1−_*_x_*As/GaAs/A1*_x_*GaAs_1−_*_x_*/GaAs	Thicknesses and AIGaAs composition	*ρ*	FE&E and EMA	Oxide (26±1)Å GaAs (435 ± 8) Å AIGaAs (413 ± 14) Å AIGaAs, *x* = 0.37 ± 0.005 GaAs (142+10) Å AIGaAs/GaAs SLS 5@ (470±20)Å AIGaAs/GaAs SLS *x* = 0.32 ± 0.001	13
SE	Al*_x_*Ga_1−_*_x_*As/GaAs	AIGaAs composition and thickness *in situ*	*ρ*	Trajectories in ∊_1_ and ∊_2_	*x* = 0.2 ± 0.03 (*x*> 0.2)	14

a Typically calculated as the 90% *c*onfidence interval from the mean square deviation and covariance matrix.

References

[1] R.M.A. Azzam and N. M. Bashara, Ellipsometry and Polarized Light, North Holland, Amsterdam (1989) Ch. 4.

[2] D. E. Aspncs, J. B. Theeten, and R. P. H. Chang, J. Vac. Sci. Technol. 16, 1374 (1979).

[3] B. J. Mrstik, P. J. McMarr, J. R. Blanco, and J. M. Bennett, J. Electrochem. Soc. 138, 1770 (1991).

[4] D. E. Aspnes and J. B. Theeten, J. Electrochem. Soc. 127, 1359 (1980).

[5] G. E. Jellison, Jr., J. Appl. Phys. 69, 7627 (1991).

[6] N. V. Nguyen and K. Vedam, J. Appl. Phys. 67, 3555 (1990).

[7] D. E. Aspnes, J. Vac. Sci. Technol. 18, 289 (1981).

[8] M. Fried, T. Lohner, W. A. M. Aarnink, L. J. Hanekamp, and A. van Silfhout, J. Appl. Phys. 71, 2835 (1992).

[9] W. M. Duncan and S. A. Henck, Appl. Surf. Sci. 63, 9 (1993).

[10] M. Fried, T. Lohner, J. M. M. de Nigs, A van Silfhout, L. J. Hanekamp, Z. Laezik, M. Q. Khanh, and J. Gyulai, J. Appl. Phys. 66, 5052 (1989).

[11] R. K. Sampson and H. Z. Massoud, J. Electrochem. Soc. 140, 2673 (1993).

[12] P. G. Snyder, M. C. Rost, G. H. Bu-Abbud, J. A. Woollam, and S. A. Alterovitz, J. Appl. Phys. 60, 3293 (1986).

[13] K. G. Merkel, P. G. Snyder, J. A. Woollam, S. A. Alterovitz, and A. K. Rai, Jpn. J. Appl. Phys. 28, 1118 (1989); J. A. Woollam, P. G. Snyder, K. G. Merkel, and S. A. Alterovitz, Materials Sci. Engl. B5, 291 (1990).

[14] D. E. Aspnes, W. E. Quinn, and S. Gregory, Appl. Phys. Lett. 56, 2569 (1969).

**Table 4:** Infrared sensitivity. Given are sensitivities for the measurement of interstitial oxygen [O_i_], substitutional carbon [C_s_], and nitrogen [Si-N-Si], in crystalline Si and substitutional carbon [C_Ga_] and interstitial oxygen [O_i_] in crystalline GaAs. Also given are sensitivities for measurement of compositions, *x*, of Al*_x_*Ga_1−_*_x_*As and Hg*_x_*Cd_1−_*_x_*Te and for carrier concentrations, *N*_d_, in Si and GaAs. The sensitivity of far infrared low-temperature absorption measurements for substitutional boron [B_s_]; phosphorus [P_s_]; and arsenic [As_s_] are also given. The notations used for the measured quantity column are absorption coefficients, *α*, at subscripted wavelength in cm^−1^, e.g., α_1107_; absorption coefficient full width product, *α∆*, at subscripted wavelength, e.g., *α∆*_845_; LO phonon frequency, *ω*_Lo_; plasma resonance frequency, *ω*_p_; transmission, *T*; and frequency, *ω*

Method	Matrix	Quantity	Measured quantity	Conversion	Sensitivity^a^	Ref. (see below)
LVM	Si	[O_i_]	α_1107_ @300 K	[O_i_] = α× 3.03 ± 0.02 × 10^17^ cm^−2^	± 2 × 10^l5^ cm^−3^	1
LVM	Si	[O_i_]	α_1107_ @300 K	[O_i_] = a × 3.14 × 10^l7^ cm^−2^	±2 × 10^15^ cm^−3^	2
LVM	Si	[C_s_]	*α*_605_ @300 K	[C_s_] = α× 1.1 × 10^17^ cm^−2^	± 2 × 10^16^ cm^−3^	3
Absorption	Si	[Si-N-Si]	α_963_ @300 K	[N_s_] = α× 1.3 × 10^17^ cm^−2^	± 2 × 10^15^ cm^−3^	4
LVM	GaAs	[C_Ga_]	*α∆*_583_ @77 K	[C_s_] = α∆× 1.1 × 10^16^ cm^−1^ [C_s_] = α⊿× 8 ± 2 × 10^15^ cm^−1^	± 2 × 10^l4^ cm^−3^	5 6
LVM	GaAs	[O_i_]	α∆_845_ @10 K	[C_s_] = α∆× 8 × 10^16^ cm^−1^	± 2 × 10^15^ cm^−3^	7
Phonon frequency	Al*_x_*Ga_1−_*_x_*As	*x*	*Ω*_LO_or *ω*_ro_ @300 K	(*ω*_Lo_/2π*c*)/cm^−1^= 292.4+70.8*x*−26.8*x*^2^−41.13*x*^3^		8
IR cut off	Hg*_x_*Cd_1−_*_x_*Te	*x*	*T vs ω*	Ref. 9		9
IR plasma frequency	Si	*N*_d_	*ω*_p_ @300 K	$\omega_{p}^{2}=4\pi N_{c}e^{2}/\epsilon m*$		10
IR plasma frequency	GaAs	*N*_d_	*ω*_p_ @300 K	$\omega_{p}^{2}=4\pi N_{c}e^{2}/\epsilon m^{*}$		11
Absorption	Si	[B_s_]	α∆_320_ @12 K	[B_s_] = α× 1.1 × 10^17^ cm^−2^	± 1.5 × 10^12^ cm^−3^	12
Absorption	Si	[P_s_]	α∆_316_ @12 K	[P_s_] = α× 1.1 × 10^17^ cm^−2^	± 1.5 × 10^12^ cm^−3^	12
Absorption	Si	[As_s_]	α∆_382_ @12K	[As_s_] = α× 1.1 × 10^17^ cm^−2^	± 1.5 × 10^12^ cm^−3^	12

a Calculated as the concentration-equivalent-of-noise assuming ±0.1% noise in transmission.

References

[1] T. lizuka, S. Takasu, M. Tajima, T. Arai, T. Nozaki, N. Inoue, and M. Watanabe, J. Electrochem. Soc. 132, 1707 (1985).

[2] A. Baghadi, W. M. Bullis, M. C. Croarkin, Yue-Zhen Li, R. I. Scaee, R. W. Series, P. Stallhofer, and M. Watanabe, J. Electrochem. Soc. 136, 2015 (1989); ASTM Standard F1188, Annual Book of ASTM Standards, 10.05 (ASTM, Philadelphia, PA (1991).

[3] R.C. Newman and J. B. Willis, J. Phys. Chem. Solids 26, 373 (1965).

[4] H. J. Stein, Appl. Phys. Lett. 47, 1339 (1985); Y. Itoh, T. Nozaki, T. Masui and T. Abe, Appl. Phys. Lett.47, 488 (1985).

[5] A. T. Hunter, H. Kimura, J. P. Baukus, H. V. Winston, and O. J. Marsh, Appl. Phys. Lett. 44, 74 (1984)

[6] M. R. Brozel, E. J. Foulkes, R. W. Series, and D. T. J. Hurle, Appl. Phys. Lett. 49, 337 (1986).

[7] M. Skowronski, S. T. Neild, and R. E. Kremer, Appl. Phys. Lett. 58, 1545 (1991).

[8] O. K. Kim and W. G. Spitzer, J. Appl. Phys. 50, 4362 (1979); S. Adachi, J. Appl. Phys. 58, R1 (1985).

[9] E. Finkman and Y. Nemirovsky, J. Appl. Phys. 50, 4356 (1979).

[10] W. G. Spitzer and H. Y. Fan, Phys. Rev. 106, 882 (1957).

[11] J. K. Kung and W. G. Spitzer, J. Electrochem. Soc. 121, 1482 (1971).

[12] S. C. Baber, Thin Solid Films 72, 201 (1980).

**Table 5:** Photoreflectance spectroscopy sensitivity. Given are sensitivities for the measurement of crystallinity, *x*; stress, *X*; field strength, *F*_dc_; surface photovoltage, *V*_f_; and doping density, *N*_d_. The notations used for the measured quantity column are intensity, *I*; energy, *hv*; damping, *Γ*; bandgap, *E*_g_; splitting energy, ∆*E*_split_; deformation potential, *b*; compliances, *S*_11_ and *S*_12_; energy of Franz Keldysh oscillation lobe, *E_m_*; oscillation number, *m*; energy difference, *E*_2_−*E*_1_; shift of critical point, δ*E*_cp_; and spacing of Franz Keldysh oscillations, ∆E_FKO_. ∆*V* is the built-in potential minus the photovoltage of the laser minus the thermal energy

Method	Matrix	Quantity	Measured quantity	Conversion	Sensitivity^a^	Ref. (see below)
PR	Si	crystallinity	*I*, *hv*, *r*	qualitative		1
PR	In*_x_*Ga_1−_*_x_*As	*x*	*E*g	E_g_/cV = 1.425−1.337*x*+0.270*x*^2^	*x* = 0 to 0.15	2
PR	GaAs/Si	stress	∆*E*_split_	*∆E*_split_ = 2*b*(*S*_11_*−S*_l2_)*χ*	*X* = (150 ± 50) MPa	3
PR	GaAs/GaAlAs	field strength	*E_m_*vs *m*	*m*π = *θ*+(4/3)[(*E_m_−E*_0_)*/hO*]^3/2^	*F*_dc_−(2 to 4) × 10^5^ V/cm	4
PR	metal/GaAs	surface photovoltage	*E_m_*vs *m*	*mπ* = 0+(4/3)[(*E_m_*−*E*_0_)/*hO*]^3/2^	*V*_F_ = (0.73 ± 0.02) V	5
PR	GaAs	doping density	*E*_2_ *−E*_1_	*N_d_* = (*∆V*)*N*_d_ = (*E*_2_*−E*_1_)^3^(3.46 *×*10^20^)cm*^−^*^3^	*N*_d_ = (1 × 10^14^ to 1 × 10^16^) cm^−3^	6
PR	GaAs	doping density	*δE*_cp_	*δE*_cp_/*δN*_d_ = (5.8 ± 0.5) × 10^−20^ cV cm^3^	*N*_d_>1 × 10^16^ cm^−3^	7
PR	GaAs	doping density	∆*E*_FKO_	$\Delta E_{\mathrm{FKO}}=\mathrm{const}.\;\times N_{d}^{1/3}$	*N*_d_ = (6 × 10^17^ to 3 × 10^18^)cm^−3^	8

a Values quoted in references below.

References

[1] A. Giordana, R. Glosser, K. Joyner, and G. Pollack, J. Electronic Mat.**20**, 949 (1991).

[2] R. E. Nahory M. A. Pollack, and J. C. DeWinter, J. Appl. Phys. 46, 775 (1975).

[3] A. Dimoulas, P. Tzanetakis, A. Georgakilas, O. J. Glembocki, and A. Christou, J. Appl. Phys. **67**, 4389 (1990); T. Kanata, H. Suzawa, M. Matsunaga, H. Takakura, Y. Hamakawa, H. Kato, and T. Nishino, Phys. Rev. B41, 2936 (1990).

[4] X. Yin, F. H. Pollak L. Pawlowiez, T. O’Neill, and M. Hafizi, Appl. Phys. Lett 56, 1278 (1990); N. Bottka, D. K. Gaskill, P.D. Wright, R. W. Kaliski, and D. A. Williams, J. Crystal Growth 107, 893 (1991).

[5] X. Yin, H.M. Chen, F. H. Pollak, Y. Chan, P. A. Montano, P. D. Kirchner, G. D. Pettit, and J. M. Woodall, Appl. Phys. Lett. **58**, 260 (1991).

[6] M. Sydor, J. Angelo, W. Mitchel, T. W. Haas, and M-Y. Yen, J. Appl. Phys. **66**, 156 (1989).

[7] L. Peters, L. Phaneuf, L. W. Kapitan, and W. M. Theis, J. Appl. Phys. **62**, 4558 (1987).

[8] W. M. Duncan and A. F. Schreiner, Solid State Commun. **31**, 457 (1979).

**Table 6:** Photolumineseenee sensitivity. Given are sensitivities for the measurement of substitutional boron, [B_s_]; phosphorus, [P_s_]; arsenic, [As_s_]; and aluminum, [Al_s_], in crystalline Si. Sensitivities for determination of ternary composition, *x*, are given for Al_a_Ga_1−a_As, In*_x_*Ga_1−_*_x_*As, and Zn*_x_*Cd_1−_*_x_*, Tc. The notations used for the measured quantity column are the boron transverse optical hl multiexeiton peak intensity, *I*(B_TO_bl); free exciton intensity, *I*(FE); phosphorus no-phonon peak intensity, *I*(P_NP_); arsenic no-phonon peak intensity, *I*(As_NP_); aluminum no-phonon peak intensity, *I*(As_NP_); and energy, *hv*

Method	Matrix	Quantity	Measured quantity	Conversion	Sensitivity^a^	Ref. (see below)
Exciton intensity ratio	Si	[B_s_]	*I*(B_TO_bl)/*I*(FE)@4.2K	log[B_s_/cm^3^] = 1.435 log[/_B_/*I*_FE_]+12.81	±2 × 10^10^ cm^−3^	1
Exciton intensity ratio	Si	[P_s_]	*I*(P_NP_)/*I*(FE) @4.2K	log[P_s_/cm^3^] = 1.280 log[*I*p/*I*_FE_]+12.79	±4 × 10^10^ cm^−3^	1
Exciton intensity ratio	Si	[As_s_]	*I*(AS_NP_)*I*(FE) @4.2K	log[As_s_/cm^3^] = 1.049 log[*I*_As_/*I*_FE_]+12.76	±1 × 10^11^ cm^−3^	1
Exciton intensity ratio	Si	[Al_s_]	*I*(Al_NP_)/*I*(FE) @4.2K	log[Al_s_/cm^3^] = 1.359 log[*I*_A1_/*I*_FE_]+13.19	±8 × 10^10^ cm^−1^	1
Peak energy	Al*_x_*Ga_1−_*_x_*As	*x*	*hv* @300 K	*hv*/cV = 1.424+1.247*x* (0 <*x*< 0.45)	± 0.002*x*	2
Peak energy	In*_x_*Ga_1−_*_x_*As	*x*	*hv* @300 K	hv/cV = 1.424−1.337*x*+0.270*x*^2^	± 0.002*x*	3
Peak energy	Zn*_x_*Cd_1−_*_x_*Te	*x*	*hv* @4.2 K	*hv/*cV = 1.605+0.505*x*+0.285*x*^2^	± 0.0002*x*	4

a Calculated as the concentration-equivalent-of noise assuming ± 0.02 signal-to-noise ratio for intensity measurements and ±2 meV and ±0.2 meV energy precision at room temparature and 4.2k, respectively.

References

[1] W. M. Duncan, M. L. Eastwood, and H.-L Tsai, Mat. Rcs. Soc. Symp. Proc. **69**, 225 (1986).

[2] H. C. Casey and M. B. Panish, Heterostructure Lasers, Part B. Materials and Operating Characteristies, Academic Press, New York (1978), p. 16.

[3] R.E. Nabory, M.A. Pollack, and J.D. DeWinter, J. Appl. Phys. **46**, 775 (1975).

[4] N. Magnea, F. Dal’bo, J. L. Pautrat, A. Million, L. Dicioccoio, and G. Fcuillet, Mat. Res. Soc. Symp. Proc. **90**, 455 (1987):W.M. Duncan, R. J. Koestncr, J. H. Trcgilgas, H.-Y. Liu, and M.-C. Chen, Mat. Res. Soe. Symp. proc. **161**.39 (1990).

**Table 7:** Raman spectroscopy sensitivity. Given are sensitivities for the measurement of stress; *σ*; crystallinity; surface damage; boron 11 concentration, [B^11^]; temperature; composition; substitutional carbon concentration, C; substitutional zinc concentration, Zn; built-in potential, *V*_bi_; and composition, *x.* The notations used for the measured quantity column arc frequency of the LO phonon, ωto; frequency of the TO phonon, ω_TO_; crystallite diameter, *L*; intensity at a given energy, *I*(2000 cm^−1^); frequency, (ω), frequency shift, ∆ω; intensity of the two LO mode, *I*(2LO); intensity of the TO mode, *I*_TO_; intensity of the LO mode, *I*_LO_or *I*(LO); intensity of the L^−^ plasmon branch, *I*(L^−^); intensity of the anti-Stokes peak, *I*_AS_; and intensity of the Stokes peak, *I*_s_

Method	Matrix	Quantity	Measured quantity	Conversion	Sensitivity^a^	Ref. (see below)
RS	Si	Stress	ω_LO_	*σ* = (2.49×10^10^Pa^−1^)×∆ω	±1 × 10^7^ Pa	1
RS	Si	Crystallinity	*L*@300K	Ref. 2		2
RS	Si	Surface damage	*I*(2000 cm^−1^)	Ref. 3		3
RS	Si	[B^11^]	*I*(620 cm^−1^)	Ref. 4		4
RS	Si	Temperature	*Ω*and ∆ω	Ref. 5		5
RS	Si, Ge_1_*_−x_*	*x*	*ω*	Ref. 6		6
RS	GaAs	Crystallinity	*L*@300K	Ref. 7		7
RS	GaAs	Crystallinity	*I*(2LO)/*I*(540) @300K	Ref. 8		8
RS	GaAs	Crystallinity	*I*_TO_/*I*_LO_ @300K	Ref. 9		9
ERS	GaAs	C_s,_ Zn_s_	*I*(148 cm^−1^), *I*(174 cm^−1^) 6K	Ref. 10	<1 × 10^15^ cm^−1^	10
RS	GaAs	*V*_bi_	*I*(LO)/*I*(L^−^)	Ref. 11	±0.2 V	11
RS	Al*_x_* Ga_1−_*_x_*As	*x*	ω_LO_	(ω_LO_/2πc)/cm^−1^= 292.4+70.8*x*−26.8*x*^2^−41.13*x*^3^	±0.01*x*	12
RS	GaAs	Temperature	ω_LO,_ω_TO_	*I*_AS_/*I*_s_	±20°C	13

a Calculated as the concentration-equivalent-of-noise or uncertainty in frequency.

*References*

[1] Th. Englert, G. Abstreitcr, and J. Pontcharra, Solid-State Eleetronies **23**, 31 (1980)

[2] H. Richter, Z. P. Wang, and L. Ley, Solid State Commun. **39**, 625, 1981; E. Bustarrct, M.A. Hachich, and M. Brunel, Appl phys Lett. **52**, 1675 (1988)

[3] J. C. Tsang, G. S. Oehrlein, I. Haller. and J. S. Custer, Appl Phys. Lctt. **46**,589 (1985).

[4] P. T. T. Wong and M. Simard-Normandin, J. Eleetrochem. Soc. 132, 980 (1985).

[5] H. Tang and I. P. Herman, Phys. Rev. **B43**, 2299 (1991).

[6] W. J. Brya, Solid State Commun. 12, 253 (1973); J. B. Rcnucci, M. A. Rcnucci.,.and M. Cardona. Solid State Commun **9**.1651 (1971).

[7] K. K. Tiong, P. M. Armitharaj, F. H. Pollak, and D. E. Aspncs, Appl.. Phys. Lctt.**44**,122(1983); P. Parayanthal and F.11. Pollak. Phys. Rev. Lett. **52**, 1822 (1984).

[8] J. Wagner and Ch. Hoffman, Appl. Phys. Lctt.**50**, 682 (1987); J. Wagner and H. Scclewind. J Appl. phys. **64**.2761 (1988).

[9] W M. Duncan, R. J. Matyi, and H.-Y Liu, Appl. Phys. Lett. 57, 163 (1990).

[10] J. Wagner, H. Seelcwind, and U. Kaufmann, Appl Phys. Lctt **51**, 1931 (1987).

[11] L A. Farrow, C. J. Sandroff, and M. C. Tamargo Appl. phys. Lctt. **51**, 1931 (1987)

[12] O. K. Kim and W. G. Spitzer, J. Appl. Phys.**50**, 4362 (1979); S. Adachi, J. Appl. Phys. **58**, RI (1985).

[13] J. R. Shealy and G. W. Wicks, Appl Phys. Lett. **50**, 1173 (1987).

